# Supplementary material for: Effect of Iodide-Based Organic Salts and Ionic Liquid Additives in Dye-Sensitized Solar Cell Performance
Source: Nanomaterials (Basel). 2022 Aug 29;12(17):2988. doi: 10.3390/nano12172988 (PMC9457700; doi:10.3390/nano12172988)
Supplement: Supplementary file 1 [file nanomaterials-12-02988-s001.zip › nanomaterials-1882455-supplementary.pdf]

Supporting Information

Effect of iodide based organic salts and ionic liquid additives in dye-sensitized solar cell performance

João Sarrato[1,†], Ana Lucia Pinto[1,†], Hugo Cruz[1,†], Noémi Jordão[1], Gabriela Malta[1], Paula S. Branco[1], J. Carlos Lima[1,*] and Luis C. Branco[1,*]

[1]LAQV-REQUIMTE, Departament of Chemistry, NOVA School of Science and Technology, FCT NOVA, Universidade NOVA de Lisboa, 2829-516, Caparica, Portugal

*Correspondence: lima@fct.unl.pt (J.C.L.); l.branco@fct.unl.pt (L.B.)

[†]These authors contributed equally to this work.

Contents

Synthesis 4

General method for the synthesis of N-ethyl-methylpyridinium iodides: 4

General method for the synthesis of N-hexyl-methylpyridinium iodides: 4

Spectra 6

^{1}H-NMR (400 MHz, $CDCl_3$) spectrum of 3-decyl-1,2-dimethylimidazolium iodide (**4**): 6

^{13}C-NMR (101 MHz, $CDCl_3$) spectrum of 3-decyl-1,2-dimethylimidazolium iodide (**4**) 6

MS spectra of 3-decyl-1,2-dimethylimidazolium iodide (**4**): 7

^{1}H-NMR (400 MHz, Methanol-d_4) spectrum of N-ethyl-3-methylpyridinium iodide (**5**): 8

^{1}H-NMR (400 MHz, Methanol-d_4) spectrum of N-ethyl-4-methylpyridinium iodide (**7**): 8

^{1}H-NMR (400 MHz, Methanol-d_4) spectrum of N-hexyl-3-methylpyridinium iodide (**6**): 9

^{13}C-NMR (101 MHz, Methanol-d_4) spectrum of N-hexyl-3-methylpyridinium iodide (**6**): 9

MS spectra of N-hexyl-3-methylpyridinium iodide (**6**): 10

^{1}H-NMR (400 MHz, Methanol-d_4) spectrum of N-hexyl-4-methylpyridinium iodide (**8**): 11

Electrochemical Impedance Spectroscopy 12

Without 4-TBP: 12

Nyquist (top) and Bode (bottom) plots of reference: 12

Nyquist (top) and Bode (bottom) plots of **E1**: 13

Nyquist (top) and Bode (bottom) plots of **E2**: 14

Nyquist (top) and Bode (bottom) plots of **E3**: 15

Nyquist (top) and Bode (bottom) plots of **E4**: 16

Nyquist (top) and Bode (bottom) plots of **E5**: 17

Nyquist (top) and Bode (bottom) plots of **E6**: 18

Nyquist (top) and Bode (bottom) plots of **E7**: 19

Nyquist (top) and Bode (bottom) plots of **E8**: 20

Nyquist (top) and Bode (bottom) plots of **E9**: 21

Nyquist (top) and Bode (bottom) plots of **E10**: 22

Nyquist (top) and Bode (bottom) plots of **E11**: 23

With 4-TBP: 24

Nyquist (top) and Bode (bottom) plots of reference: 24

Nyquist (top) and Bode (bottom) plots of **E1**: 25

Nyquist (top) and Bode (bottom) plots of **E2**: 26

Nyquist (top) and Bode (bottom) plots of **E3**: 27

Nyquist (top) and Bode (bottom) plots of **E4**: 28

Nyquist (top) and Bode (bottom) plots of **E5**: 29

Nyquist (top) and Bode (bottom) plots of **E6**: 30

Nyquist (top) and Bode (bottom) plots of **E7**: 31
Nyquist (top) and Bode (bottom) plots of **E8**: 32
Nyquist (top) and Bode (bottom) plots of **E9**: 33
Nyquist (top) and Bode (bottom) plots of **E10**: 34
Nyquist (top) and Bode (bottom) plots of **E11**: 35

Synthesis

General method for the synthesis of N-ethyl-methylpyridinium iodides:

A mixture of methylpyridine, iodoethane (1.2 eq) and 10 mL of acetonitrile was heated to 60˚C in the microwave equipment with stirring until completion. After evaporating the solvent, the product was washed 5 times with diethyl ether and dried.

N-ethyl-3-methylpyridinium iodide (**5**):

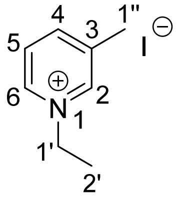

Starting from 10 g (107.4 mmol) of 3-methylpyridine and 20.1 g of iodoethane, 24.8 g (92.7%) of N-ethyl-3-methylpyridinium iodide were obtained.

^{1}H NMR (400 MHz, Methanol-*d_4*) δ (ppm) 8.94 (s, 1H), 8.86 (d, *J* = 6.1 Hz, 1H), 8.44 (d, *J* = 8.0 Hz, 1H), 8.01 (t, *J* = 7.2 Hz, 1H), 4.67 (q, *J* = 7.4 Hz, 2H), 2.61 (s, 3H), 1.67 (t, *J* = 7.4 Hz, 3H).

N-hexyl-4-methylpyridinium iodide (**7**):

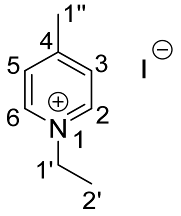

Starting from 8.5 g (91.3 mmol) of 4-methylpyridine and 17.1 g of iodoethane, 21.8 g (95.9%) of N-ethyl-4-methylpyridinium iodide were obtained.

^{1}H NMR (400 MHz, Methanol-*d_4*) δ (ppm) 8.89 (d, J = 6.3 Hz, 2H), 7.98 (d, J = 6.1 Hz, 2H), 4.67 (q, J = 7.4 Hz, 2H), 2.72 (s, 3H), 1.67 (t, J = 7.4 Hz, 3H).

General method for the synthesis of N-hexyl-methylpyridinium iodides:

A mixture of methylpyridine and iodohexane (1.2 eq) was heated to 80˚C in the microwave equipment with stirring until completion. The product was washed 5 times with diethyl ether and dried.

N-hexyl-3-methylpyridinium iodide (**6**):

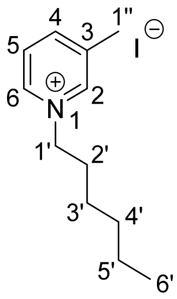

Starting from 10 g (91.3 mmol) of 3-methylpyridine and 27.2 g of iodohexane, 31.6 g (96.4%) of N-hexyl-3-methylpyridinium iodide were obtained.

^{1}H NMR (400 MHz, Methanol-*d_4*) δ (ppm) 8.97 (s, 1H), 8.87 (d, J = 6.1 Hz, 1H), 8.45 (d, J = 8.1 Hz, 1H), 8.02 (t, J = 7.1 Hz, 1H), 4.63 (t, J = 7.7 Hz, 2H), 2.61 (s, 3H), 2.05 (p, J = 7.3 Hz, 2H), 1.41 (d, J = 10.4 Hz, 6H), 1.00 – 0.90 (m, 3H).

^{13}C NMR (101 MHz, Methanol-*d_4*) δ (ppm) 147.28, 145.54, 143.06, 141.29, 128.74, 62.93, 32.42, 32.26, 26.85, 23.44, 18.49, 14.24

N-hexyl-4-methylpyridinium iodide (**8**):

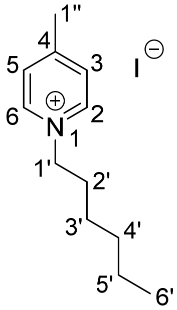

Starting from 9 g (96.6 mmol) of 4-methylpyridine and 24.6 g of iodohexane, 27.9 g (94.6%) of N-hexyl-4-methylpyridinium iodide were obtained.

^{1}H NMR (400 MHz, Methanol-*d_4*) δ (ppm) 8.90 – 8.81 (m, 2H), 7.96 (d, J = 6.2 Hz, 2H), 4.59 (t, J = 7.6 Hz, 2H), 2.71 (s, 3H), 2.02 (t, J = 7.3 Hz, 2H), 1.38 (dq, J = 10.5, 6.4, 6.0 Hz, 6H), 0.96 – 0.91 (m, 3H).

Spectra

^{1}H-NMR (400 MHz, $CDCl_3$) spectrum of 3-decyl-1,2-dimethylimidazolium iodide (**4**):

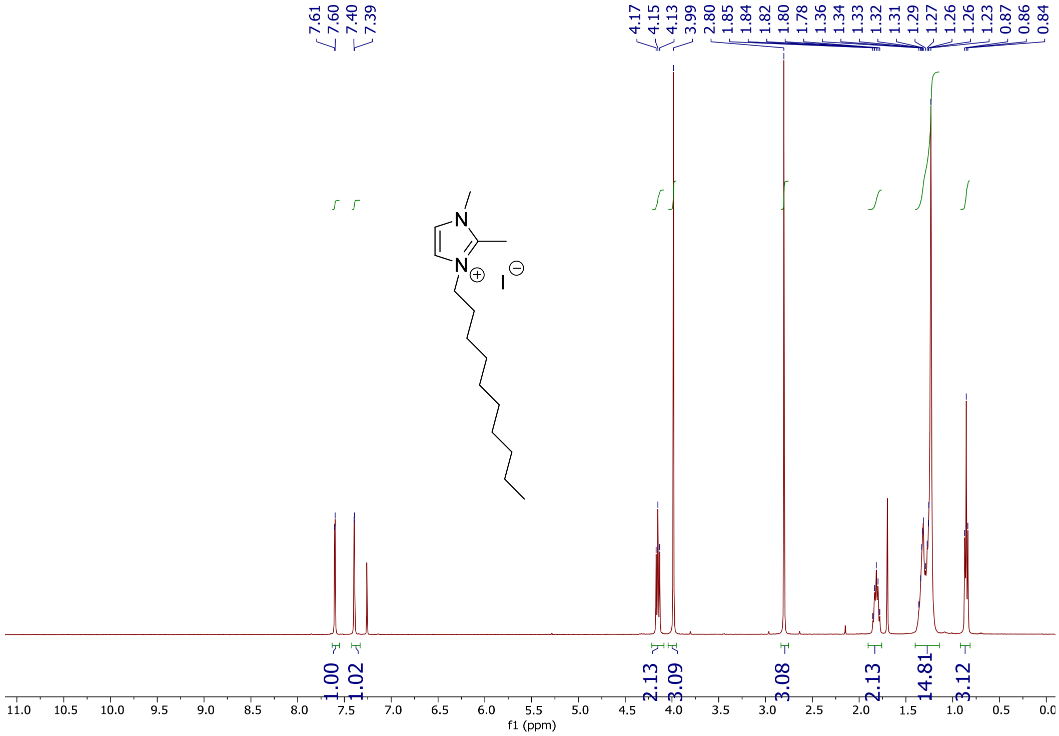

^{13}C-NMR (101 MHz, $CDCl_3$) spectrum of 3-decyl-1,2-dimethylimidazolium iodide (**4**)

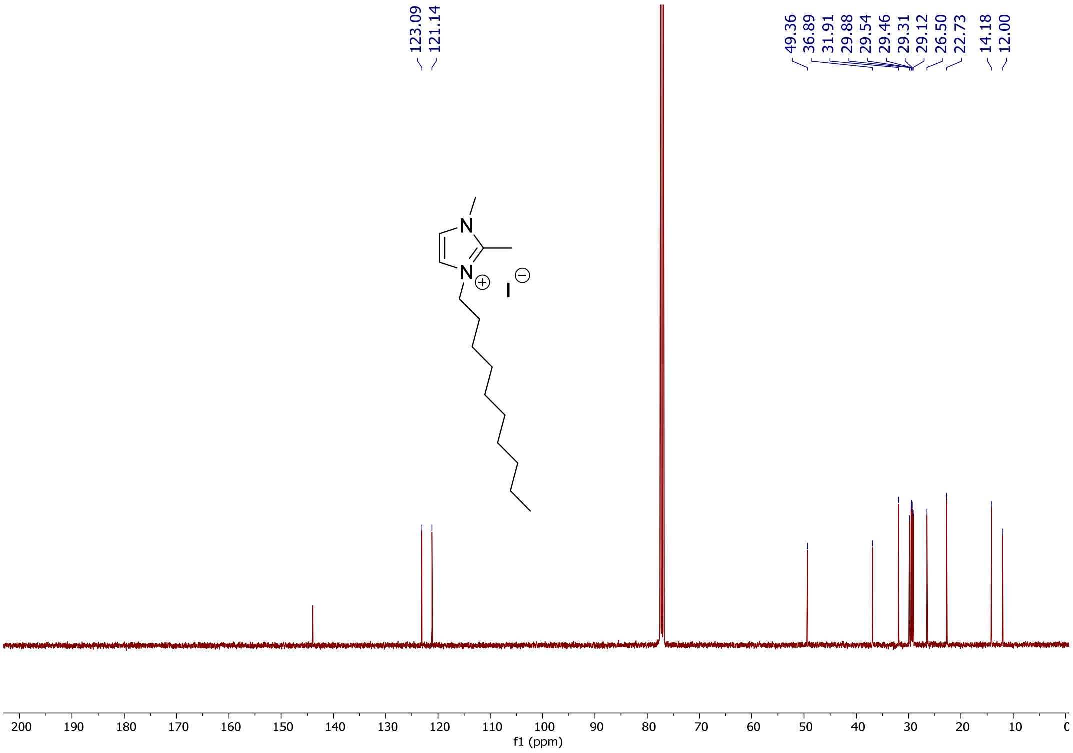

MS spectra of 3-decyl-1,2-dimethylimidazolium iodide (**4**):

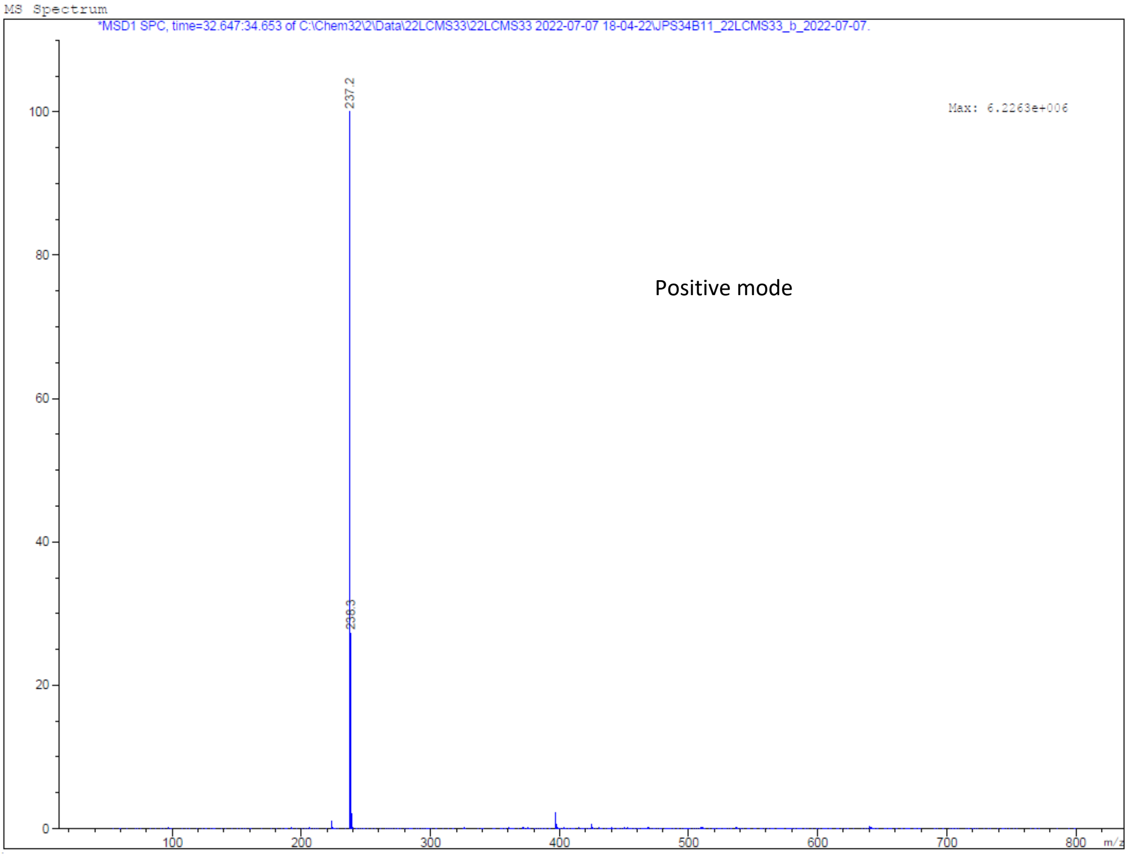

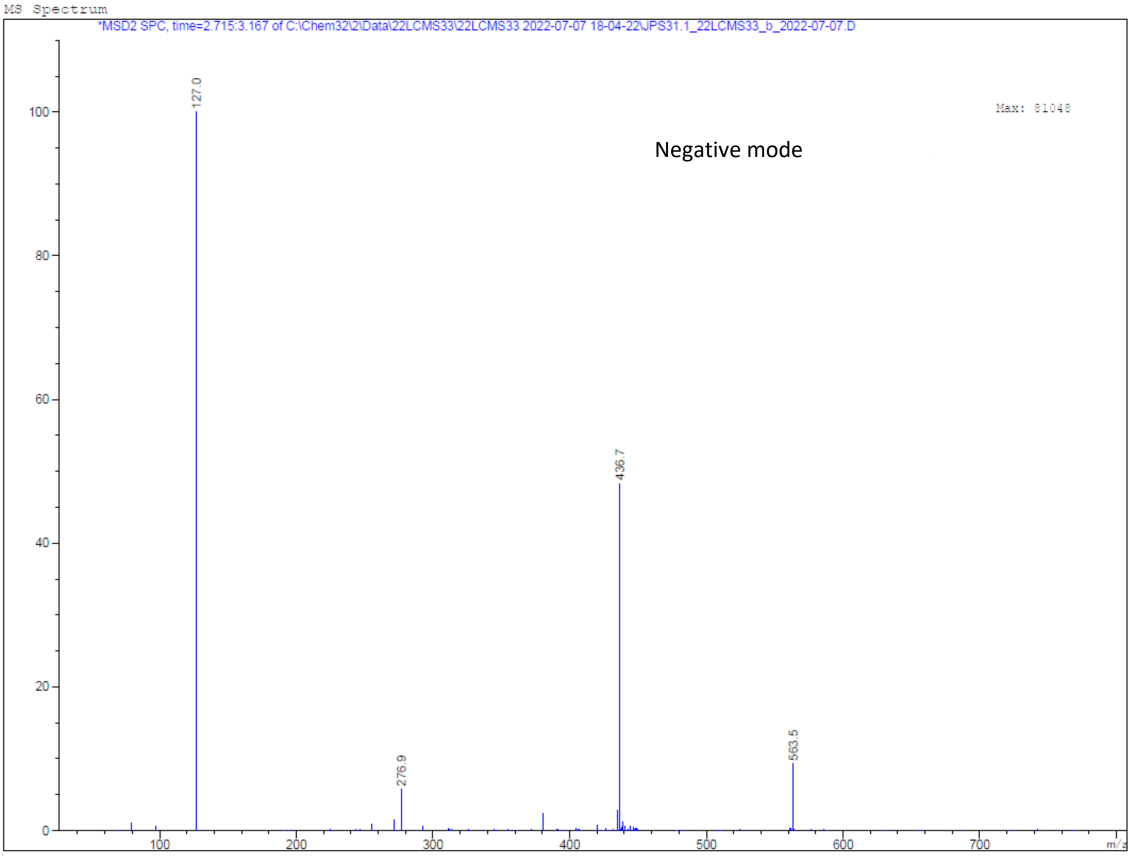

^{1}H-NMR (400 MHz, Methanol-d_4) spectrum of N-ethyl-3-methylpyridinium iodide (5):

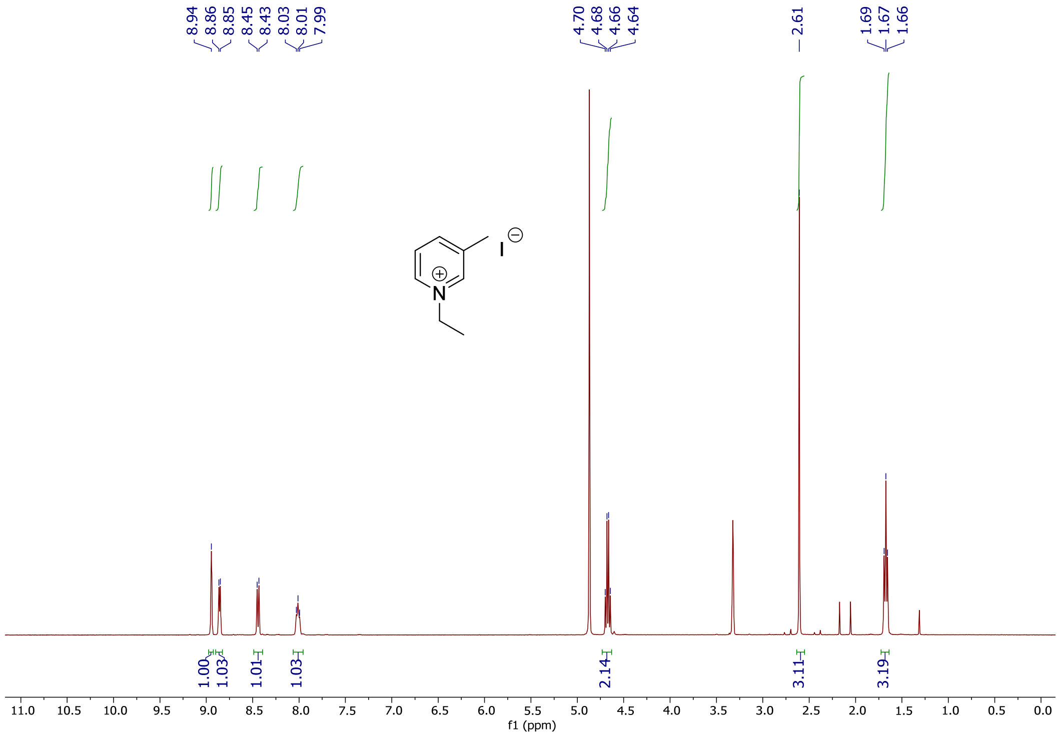

^{1}H-NMR (400 MHz, Methanol-d_4) spectrum of N-ethyl-4-methylpyridinium iodide (7):

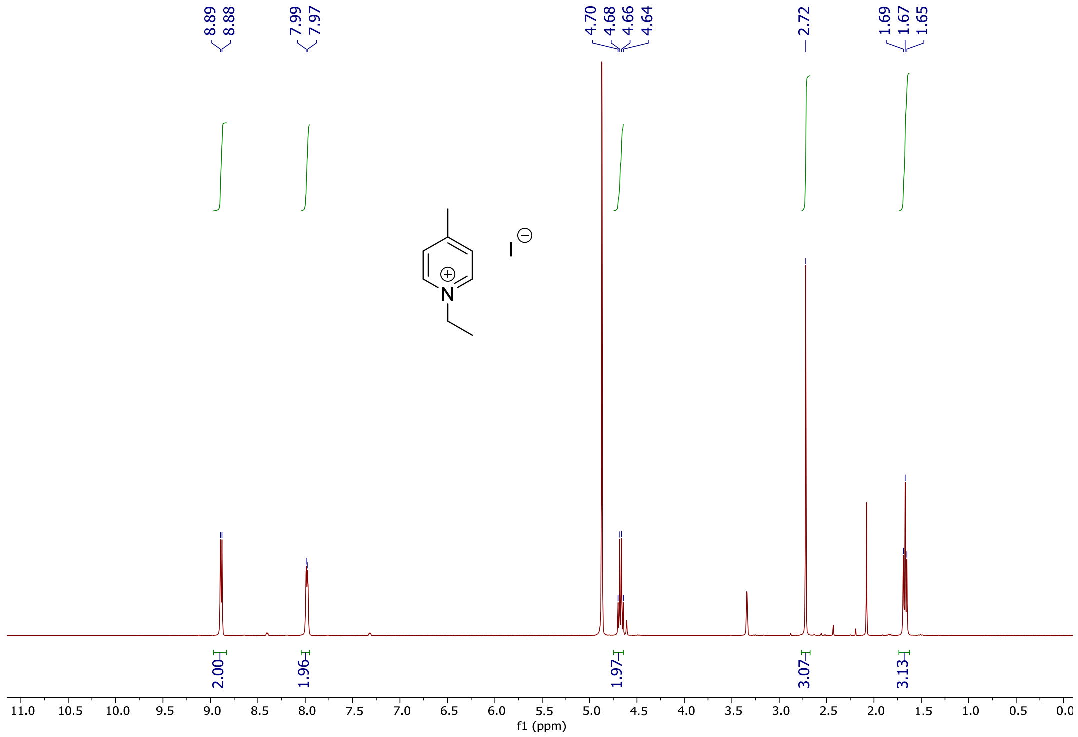

^{1}H-NMR (400 MHz, Methanol-d_4) spectrum of N-hexyl-3-methylpyridinium iodide (**6**):

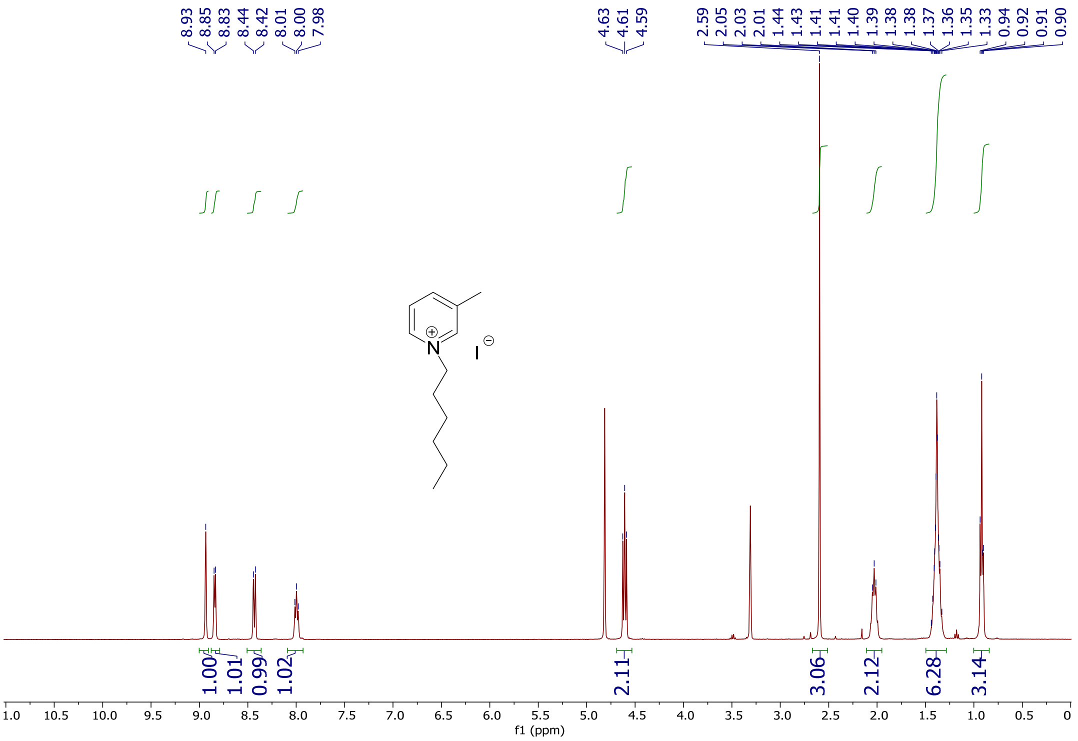

^{13}C-NMR (101 MHz, Methanol-d_4) spectrum of N-hexyl-3-methylpyridinium iodide (**6**):

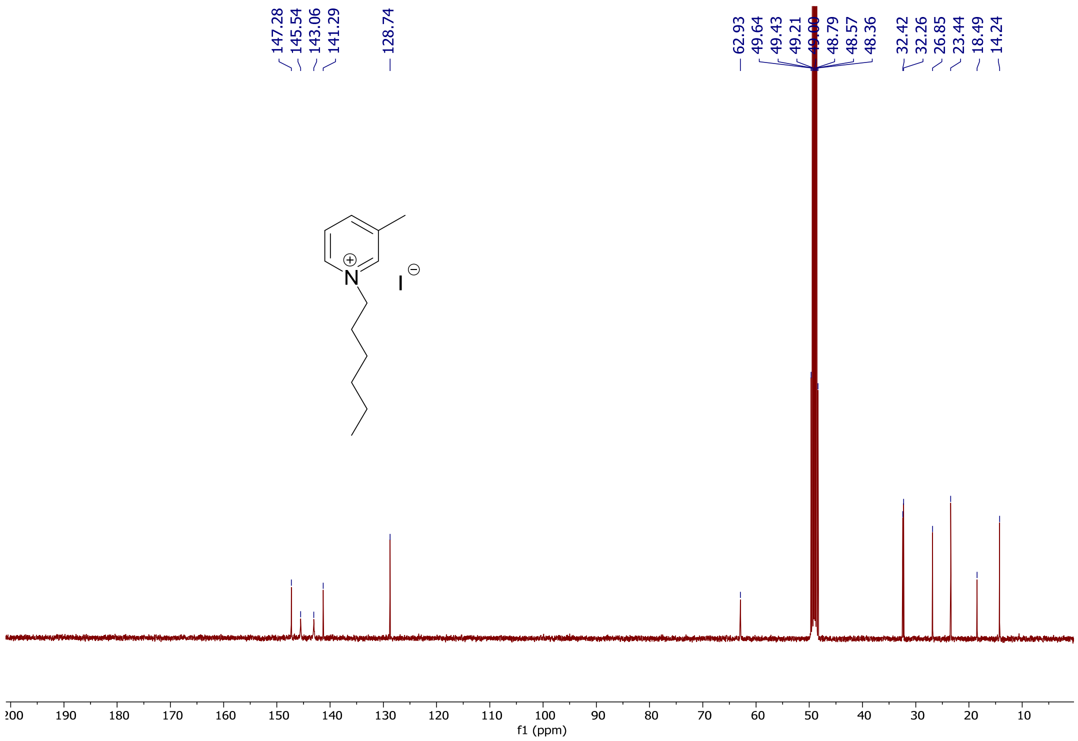

MS spectra of N-hexyl-3-methylpyridinium iodide (**6**):

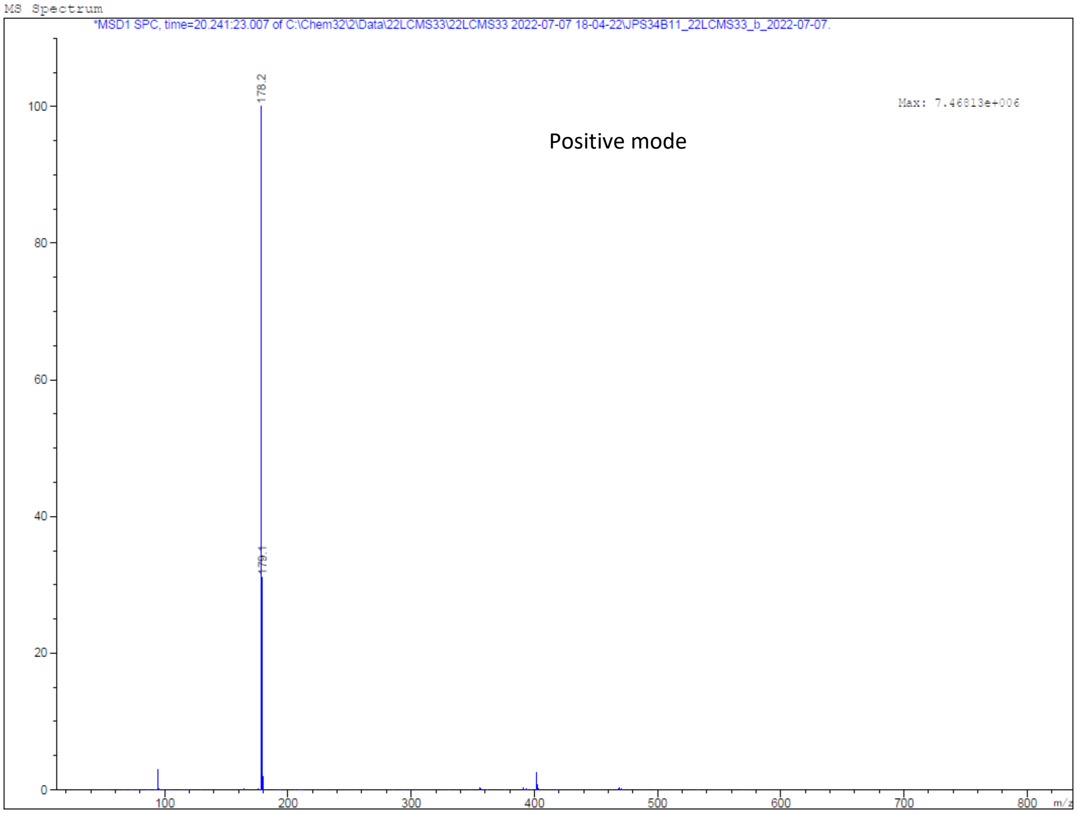

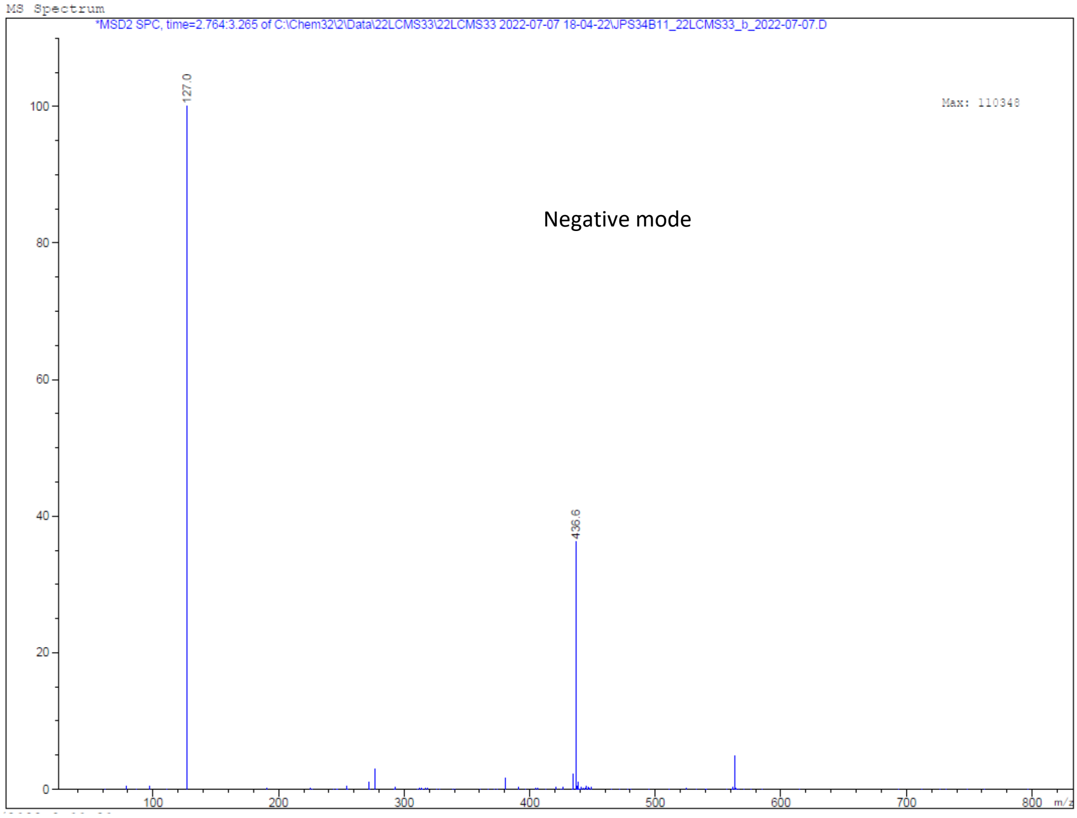

^{1}H-NMR (400 MHz, Methanol-d_4) spectrum of N-hexyl-4-methylpyridinium iodide (**8**):

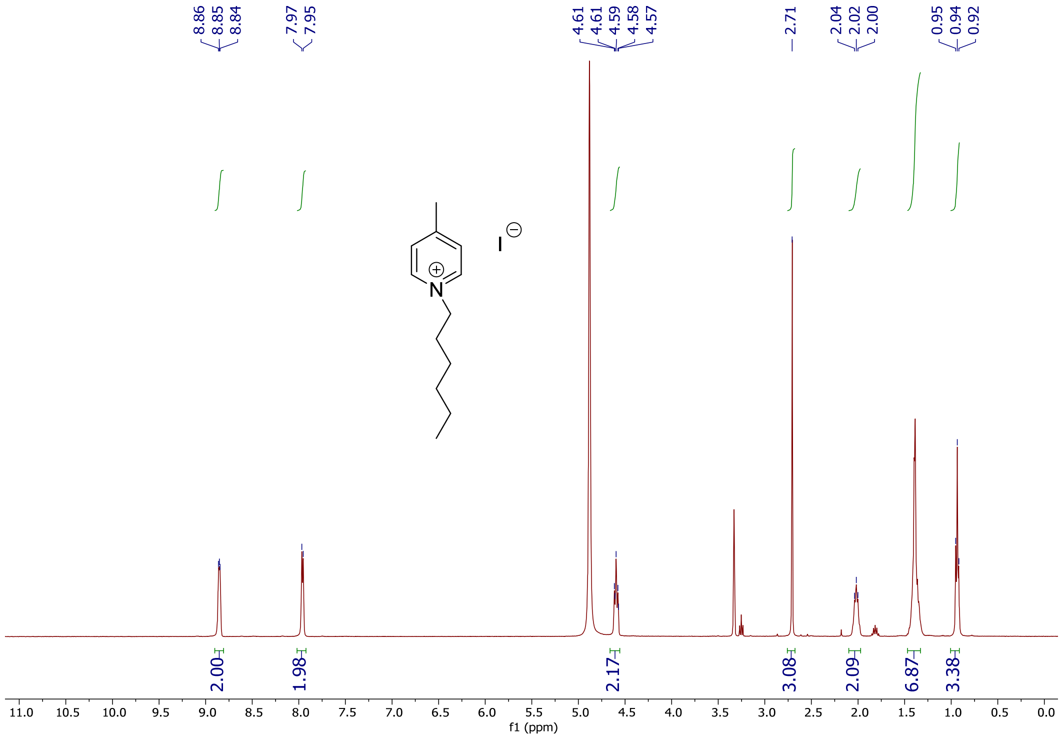

Electrochemical Impedance Spectroscopy

Without 4-TBP:

Nyquist (top) and Bode (bottom) plots of reference:

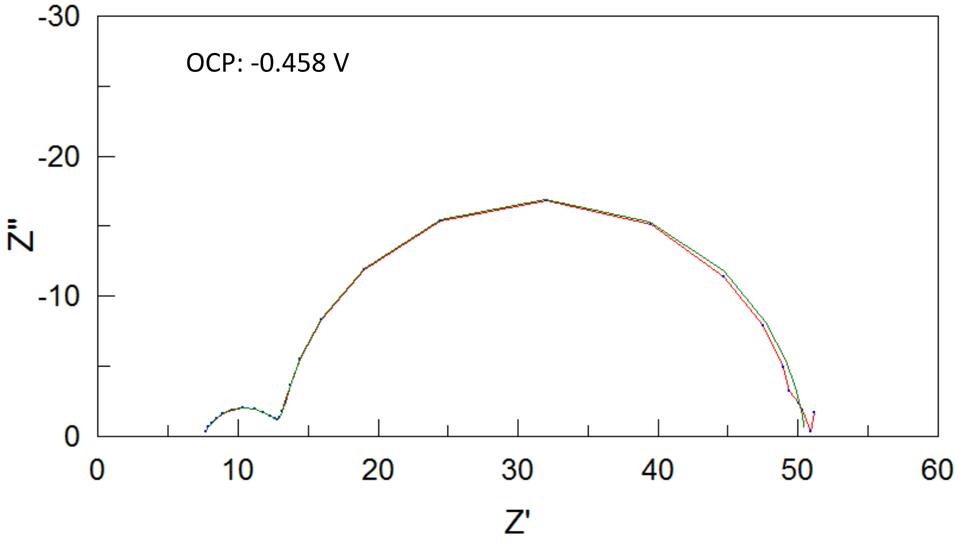

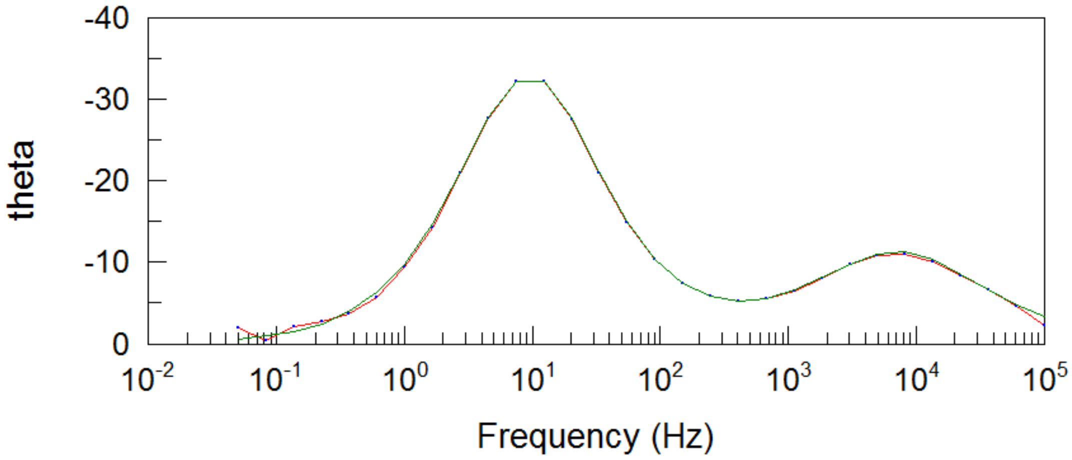

Nyquist (top) and Bode (bottom) plots of **E1**:

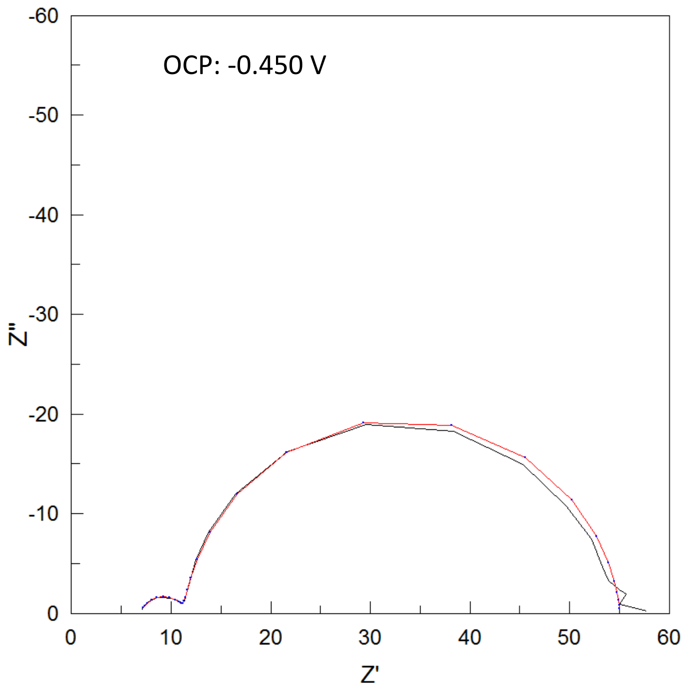

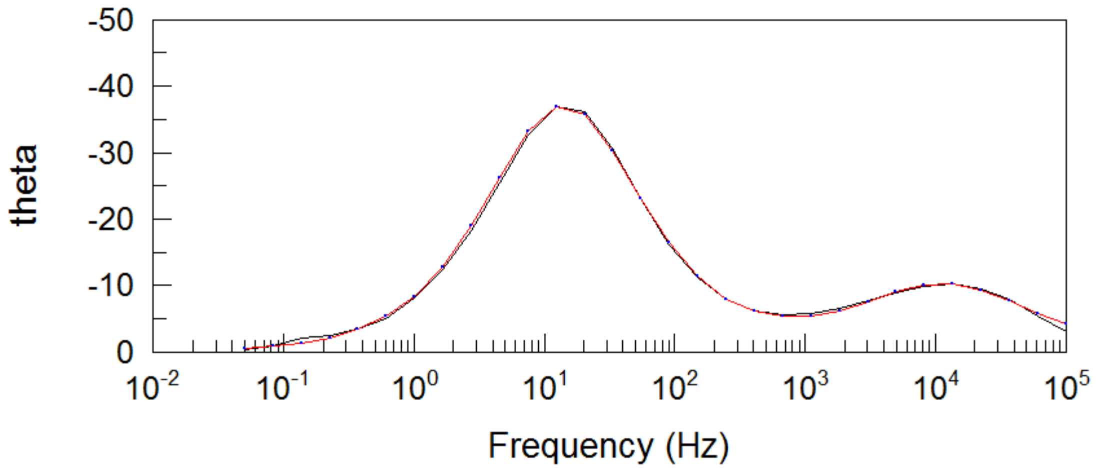

Nyquist (top) and Bode (bottom) plots of **E2**:

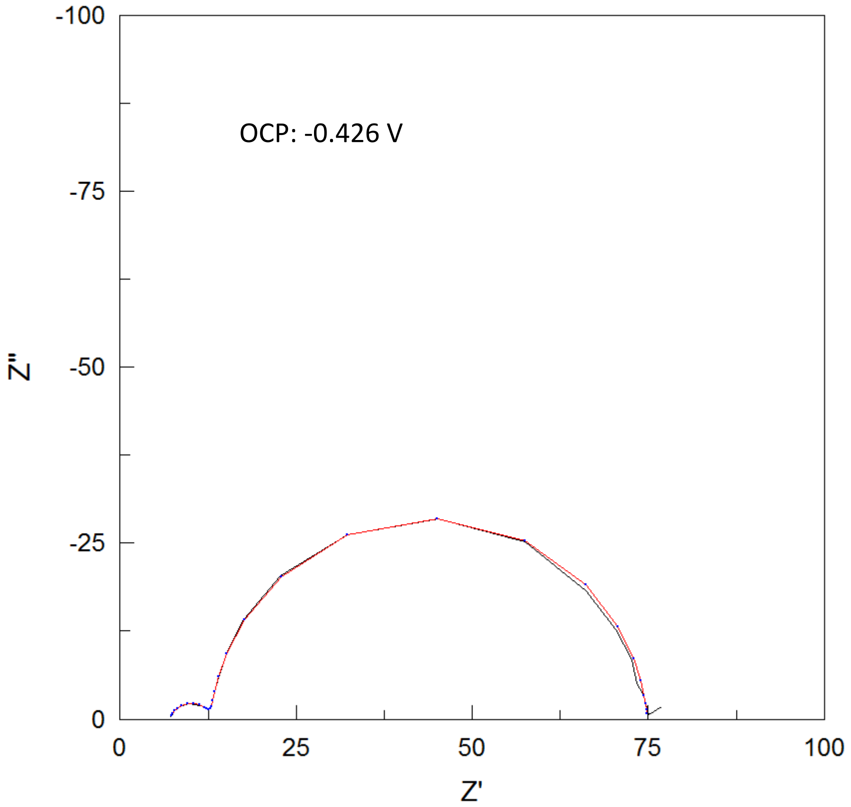

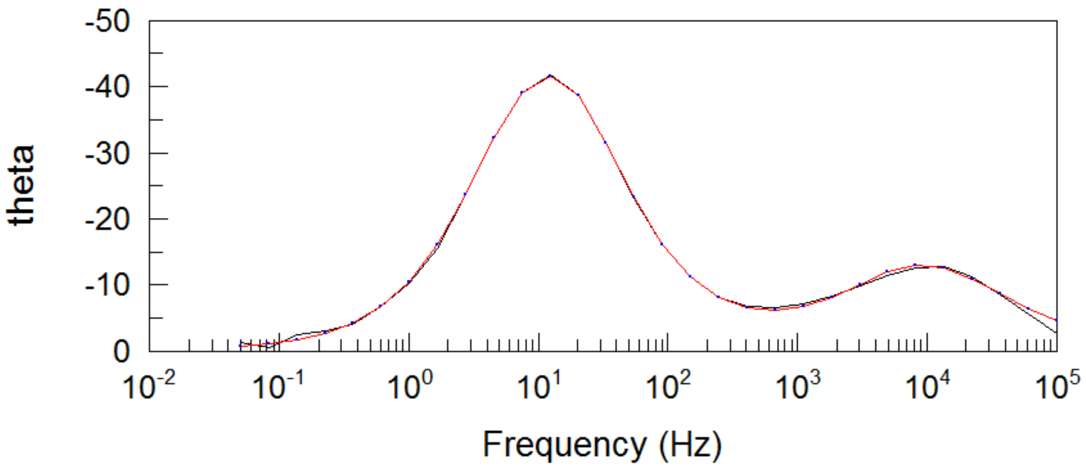

Nyquist (top) and Bode (bottom) plots of E3:

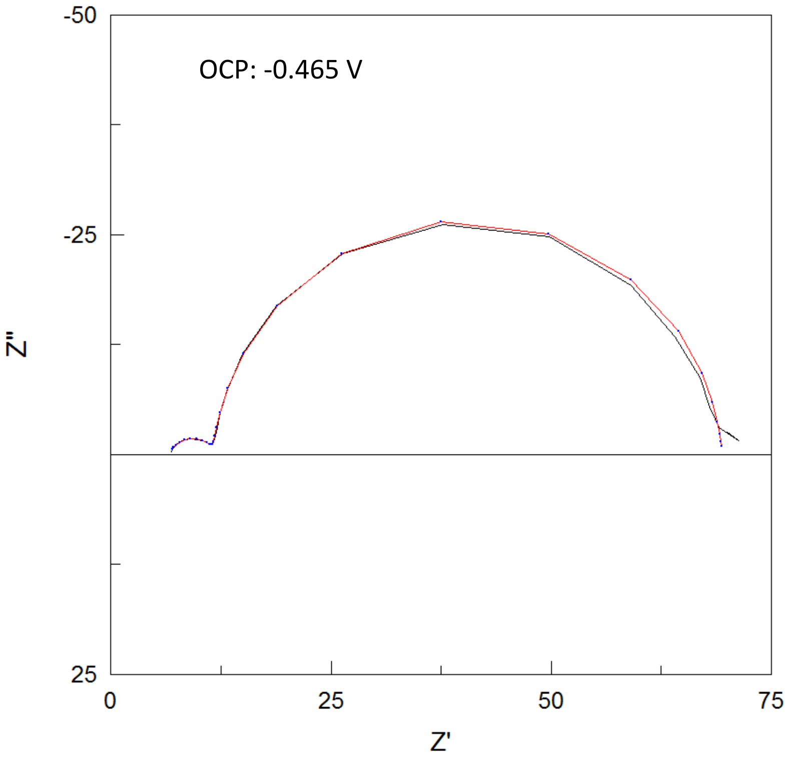

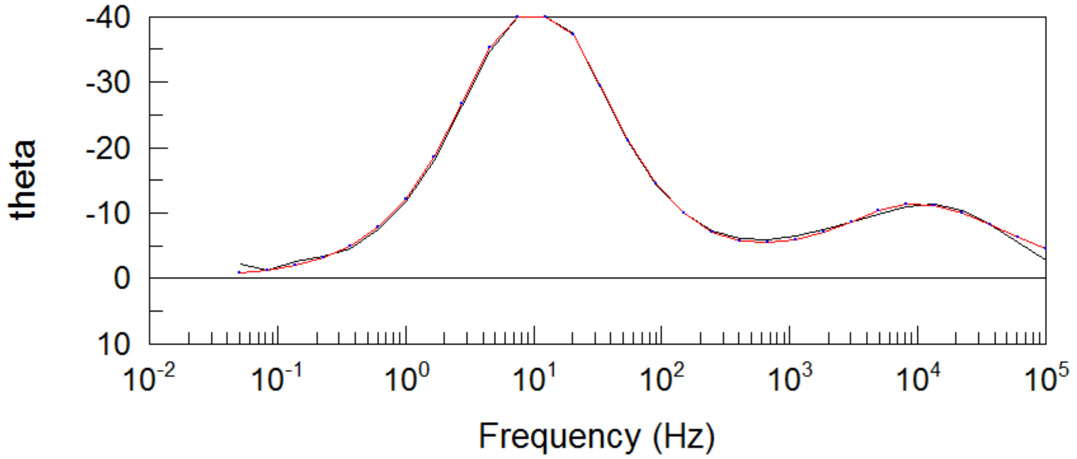

Nyquist (top) and Bode (bottom) plots of **E4**:

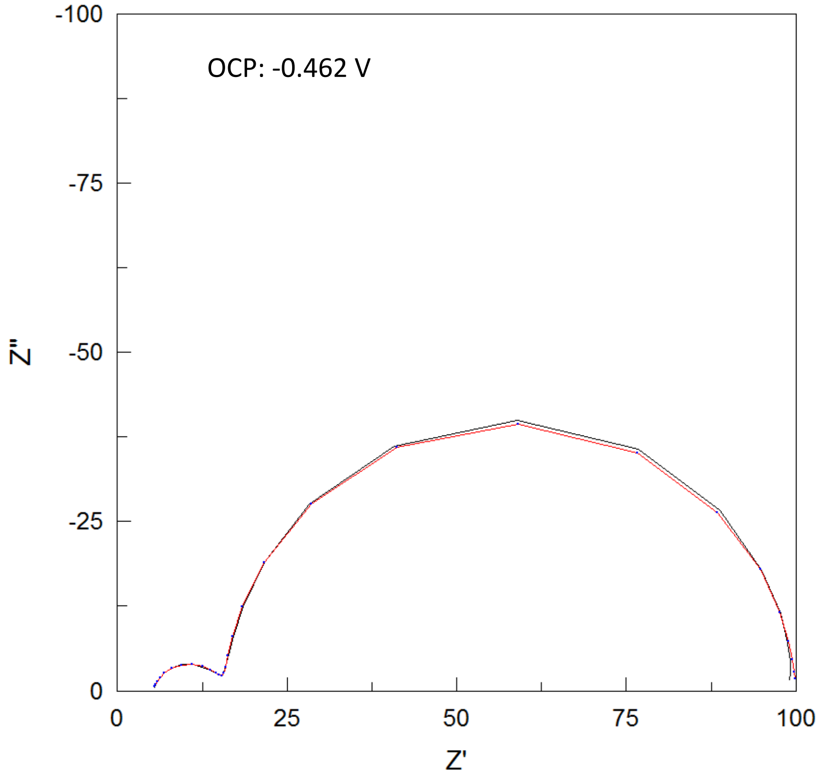

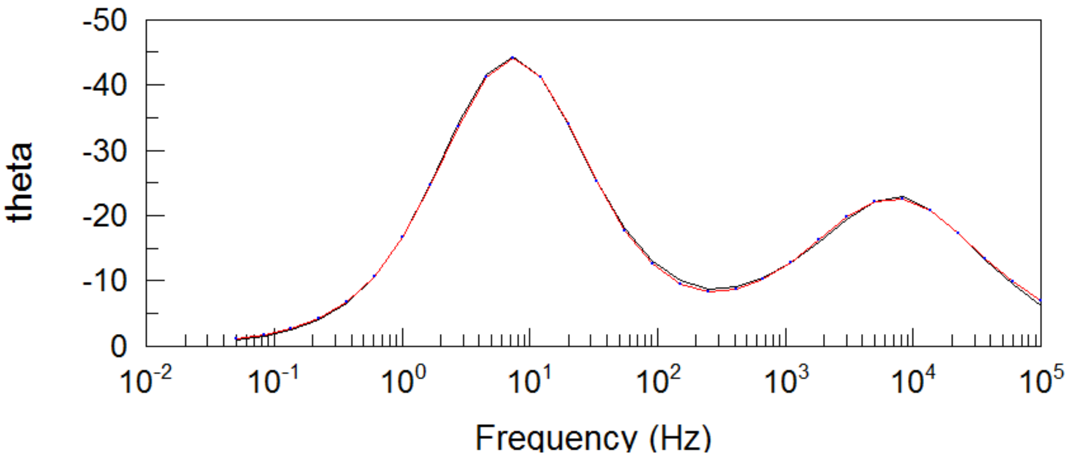

Nyquist (top) and Bode (bottom) plots of **E5**:

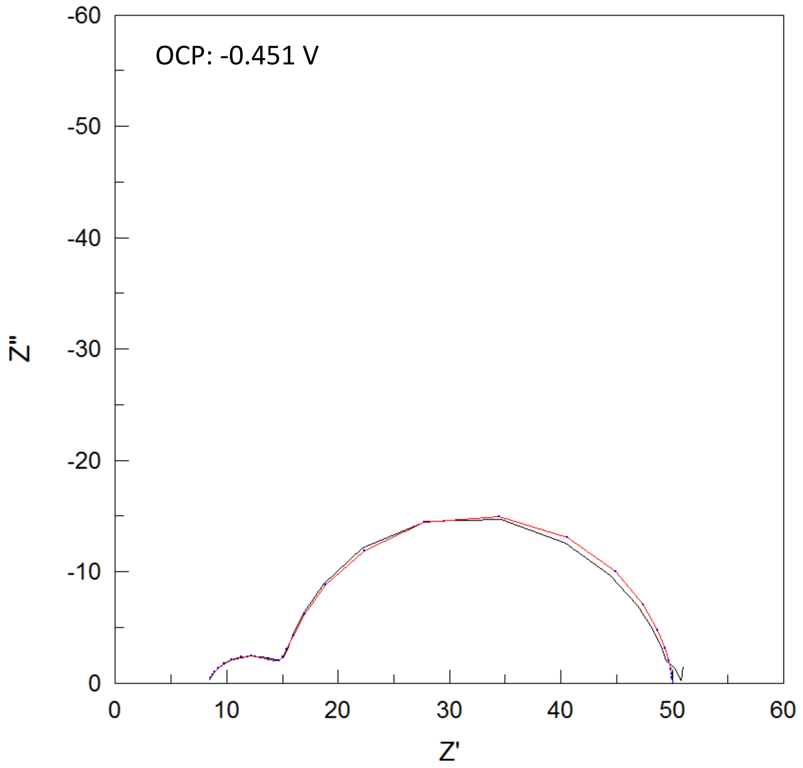

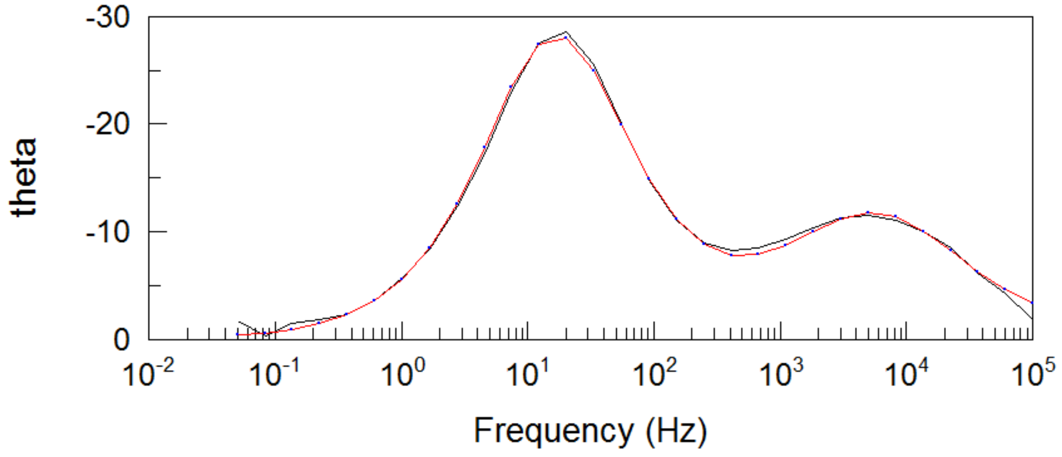

Nyquist (top) and Bode (bottom) plots of E6:

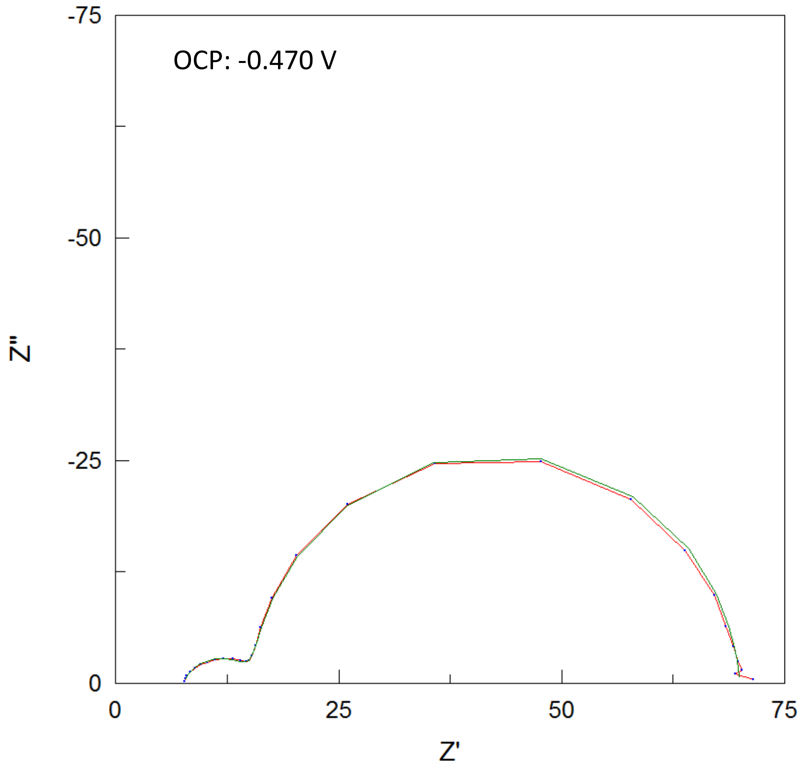

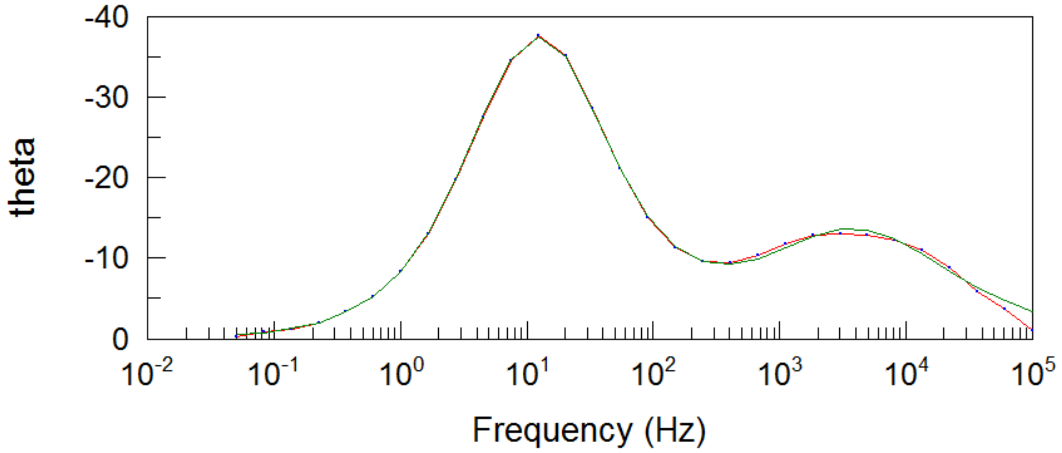

Nyquist (top) and Bode (bottom) plots of **E7**:

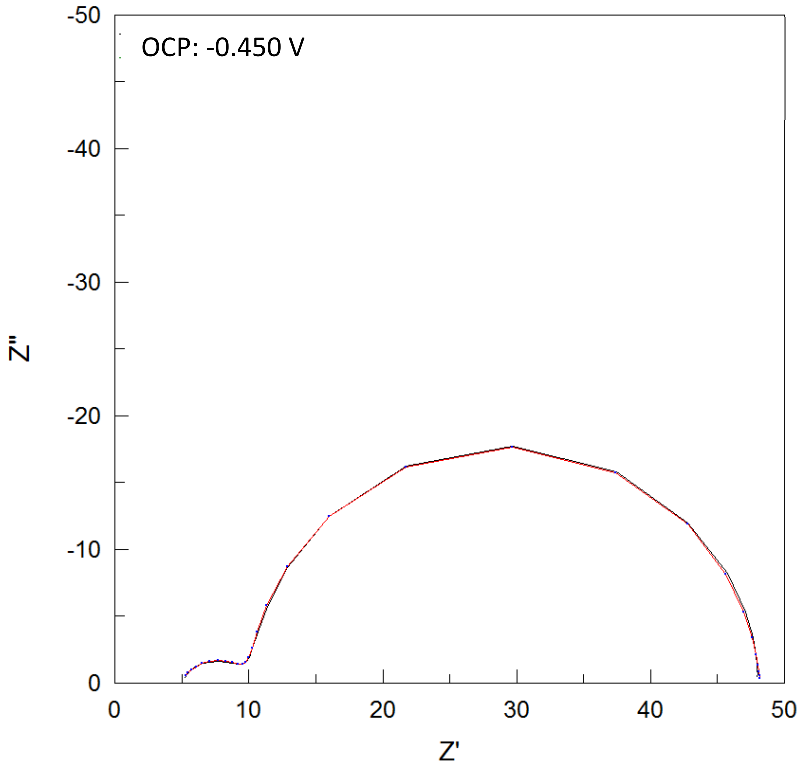

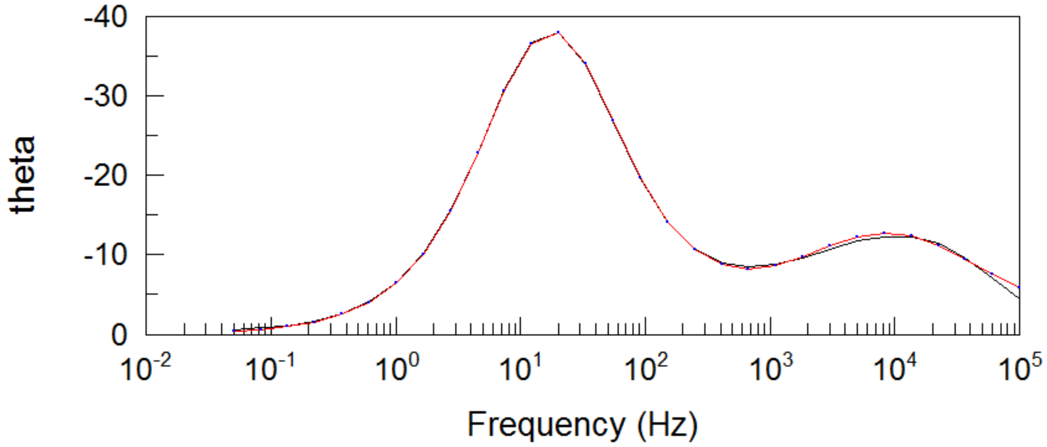

Nyquist (top) and Bode (bottom) plots of **E8**:

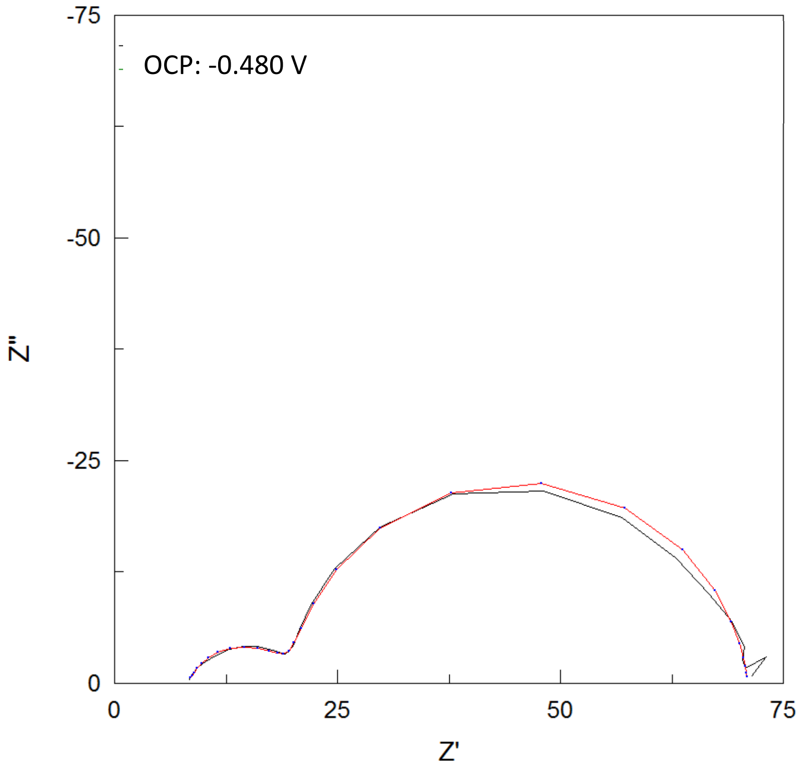

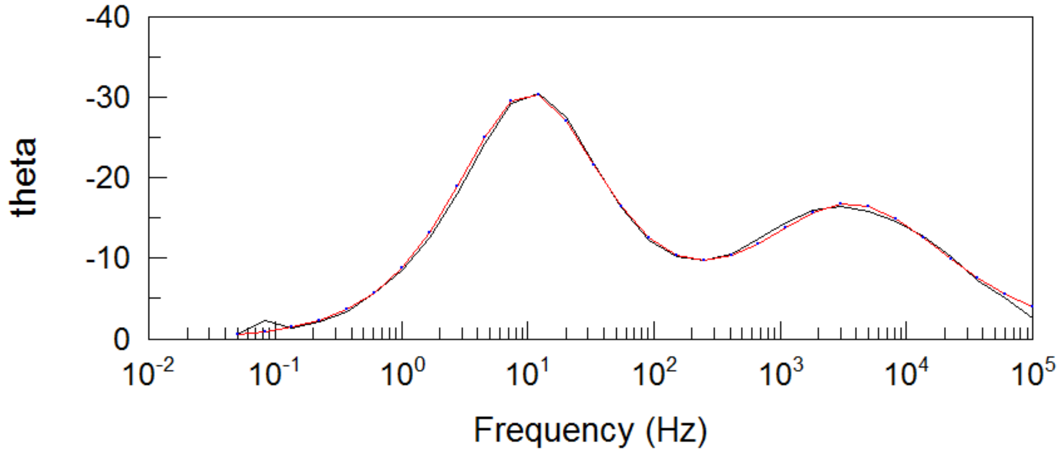

Nyquist (top) and Bode (bottom) plots of E9:

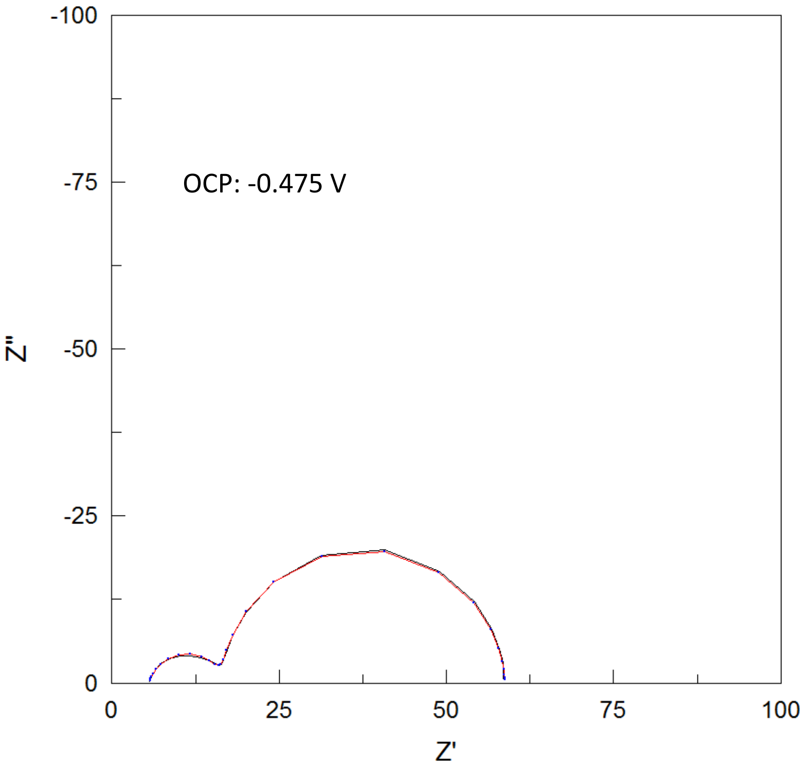

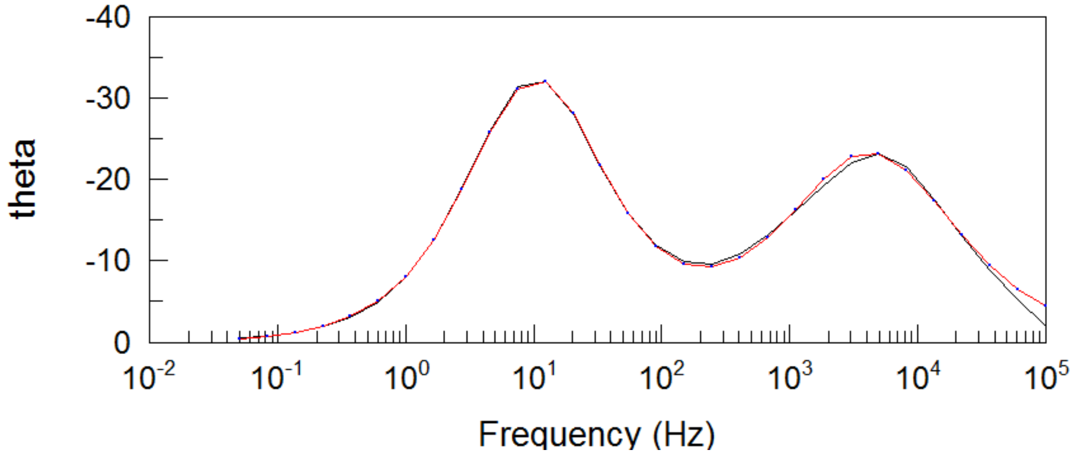

Nyquist (top) and Bode (bottom) plots of **E10**:

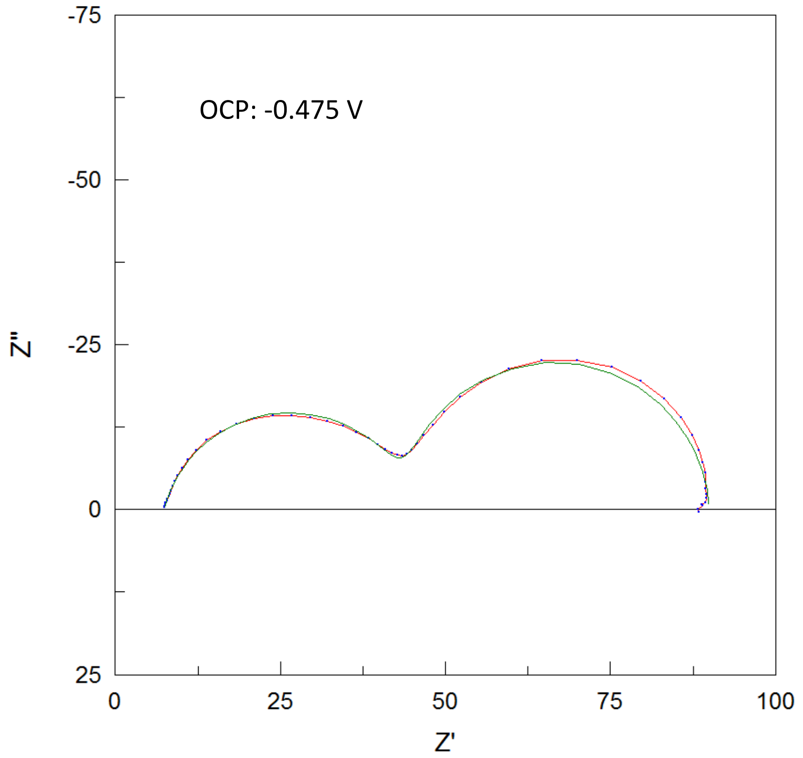

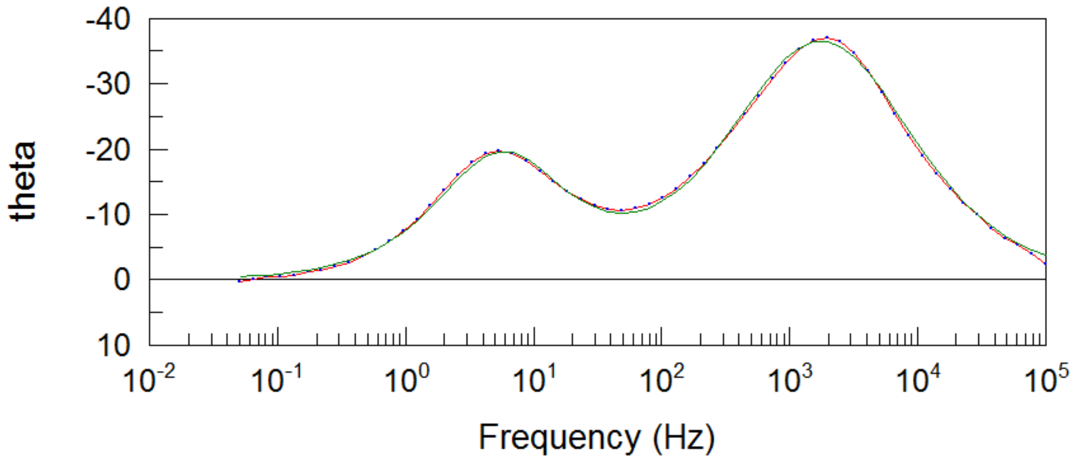

Nyquist (top) and Bode (bottom) plots of **E11**:

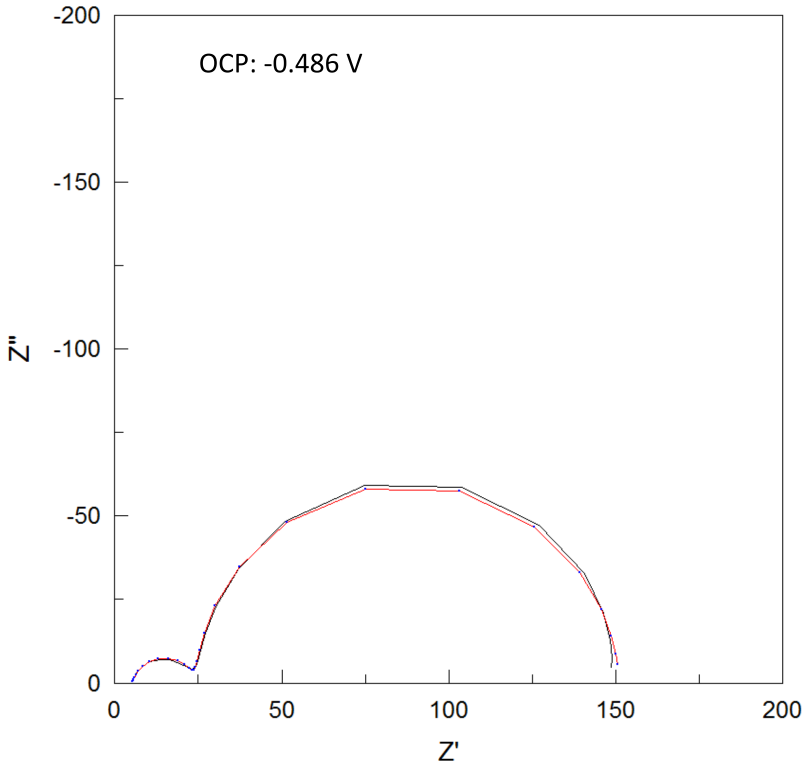

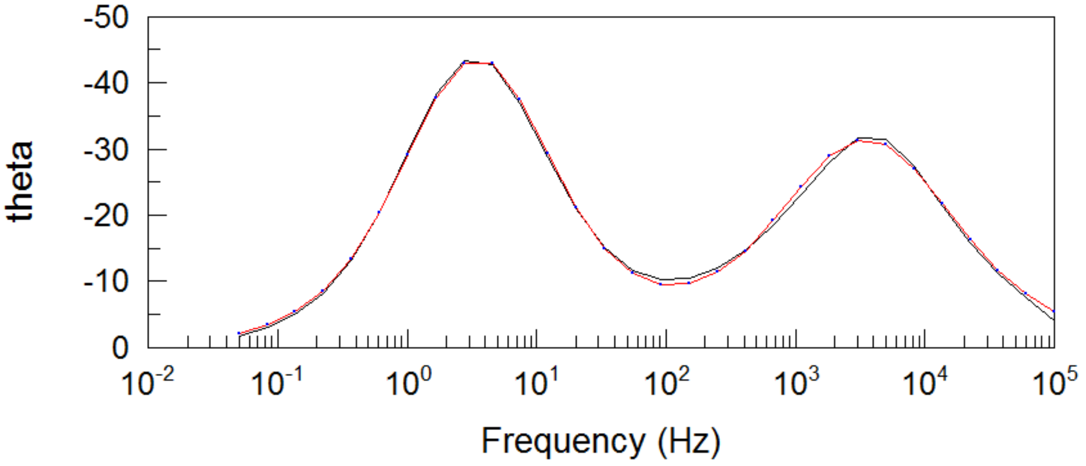

With 4-TBP:

Nyquist (top) and Bode (bottom) plots of reference:

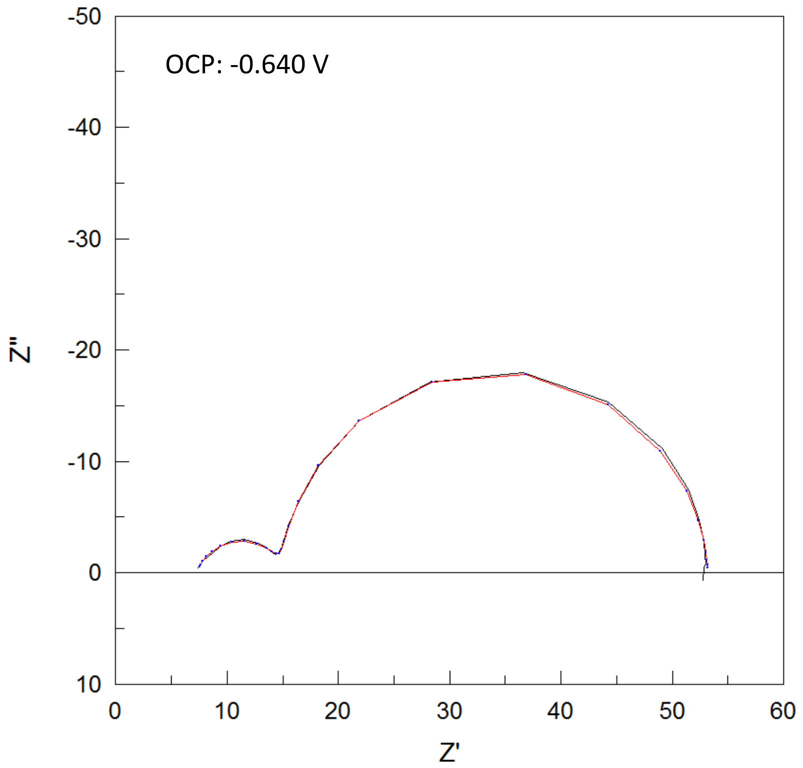

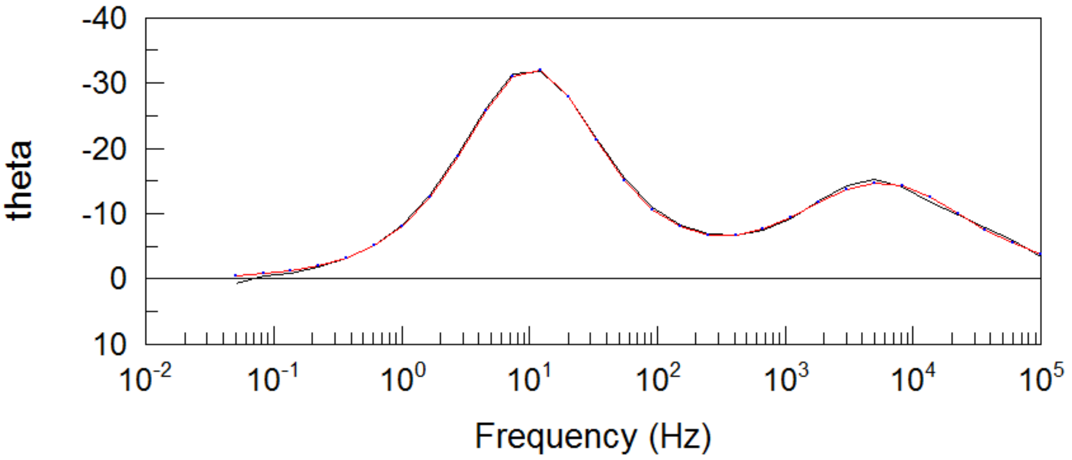

Nyquist (top) and Bode (bottom) plots of **E1**:

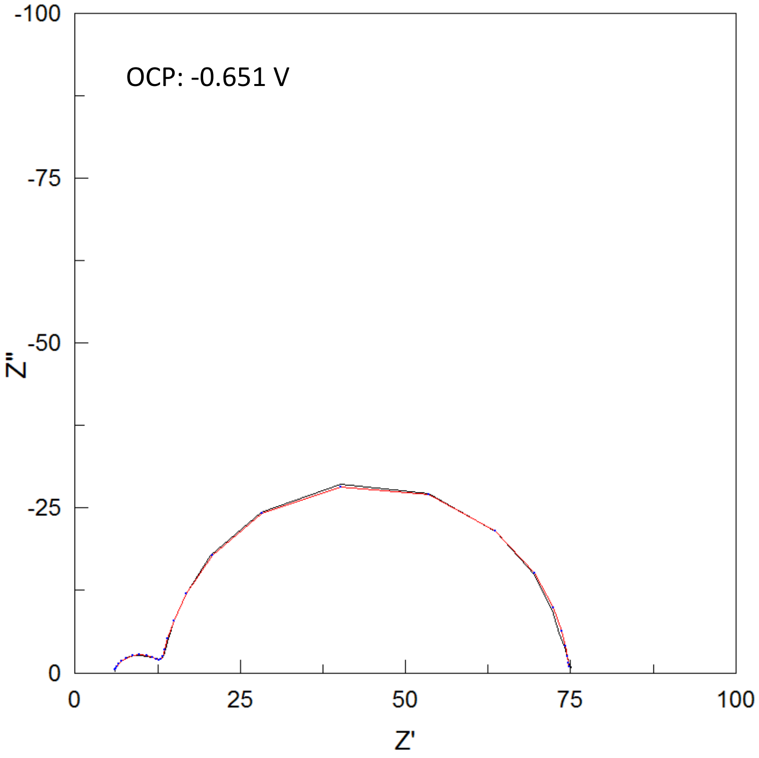

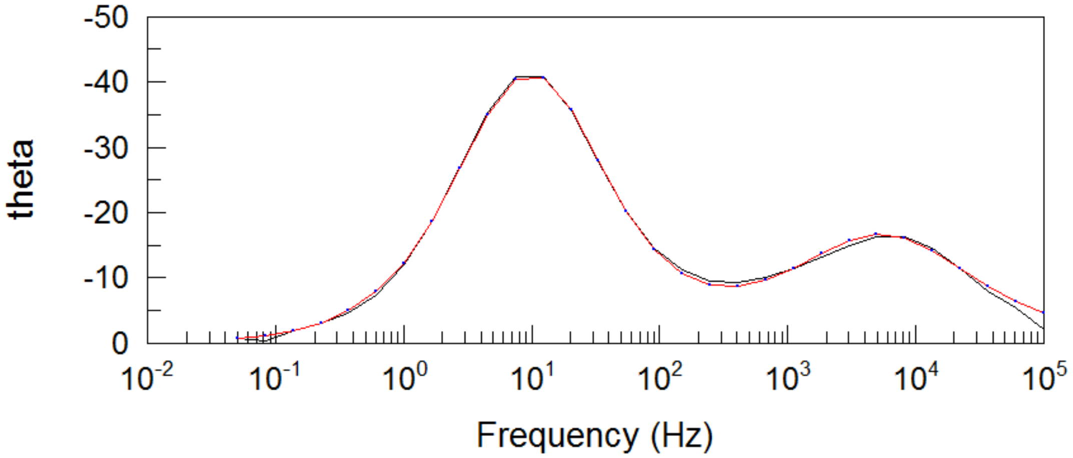

Nyquist (top) and Bode (bottom) plots of **E2**:

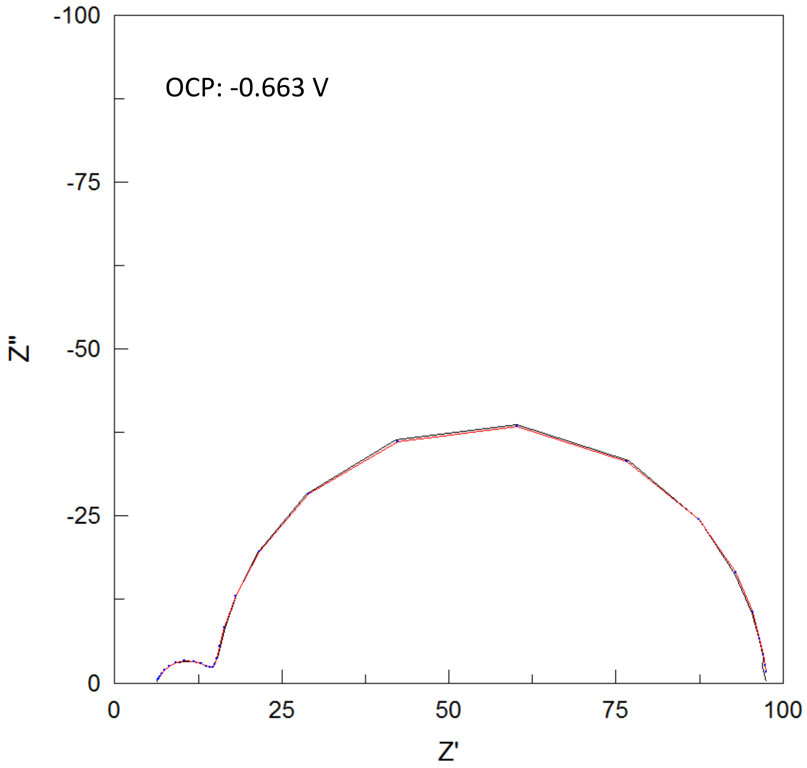

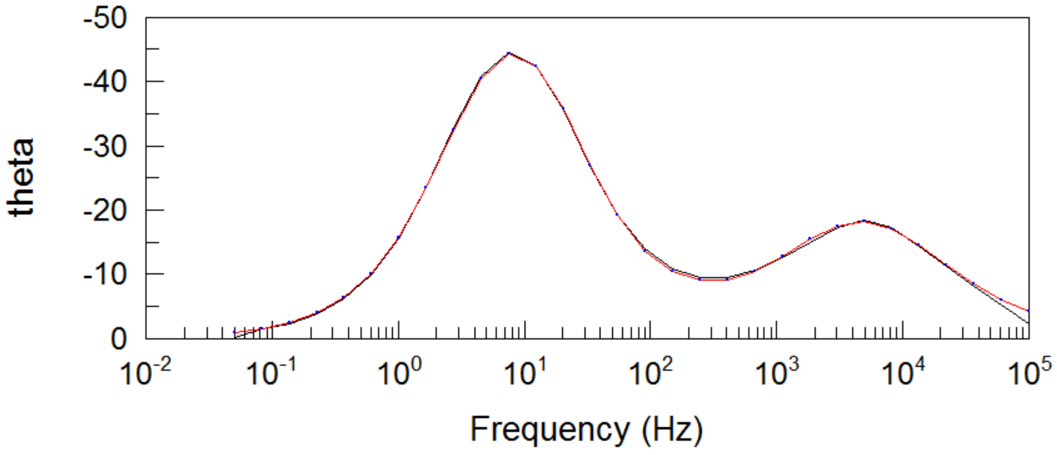

Nyquist (top) and Bode (bottom) plots of **E3**:

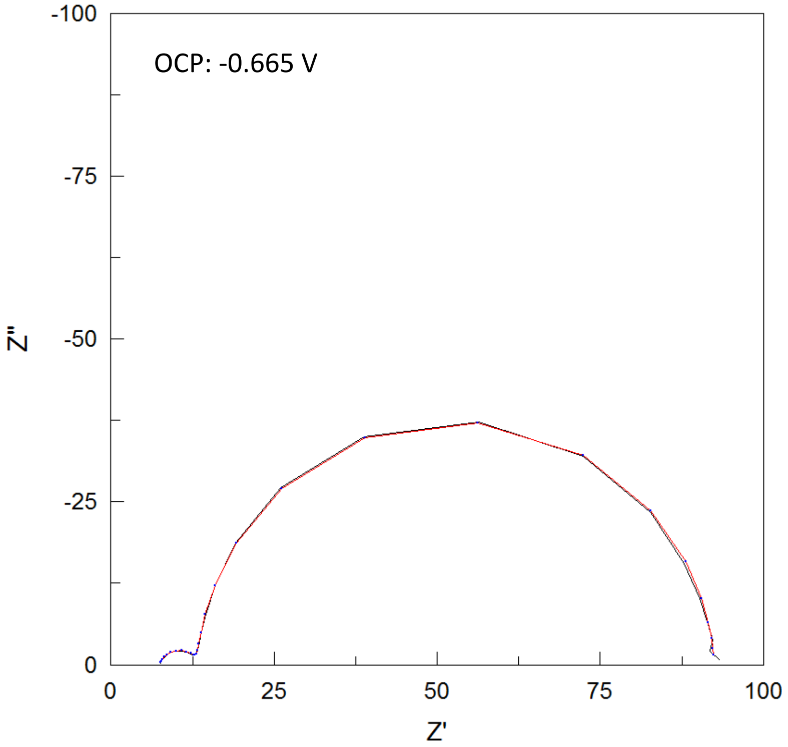

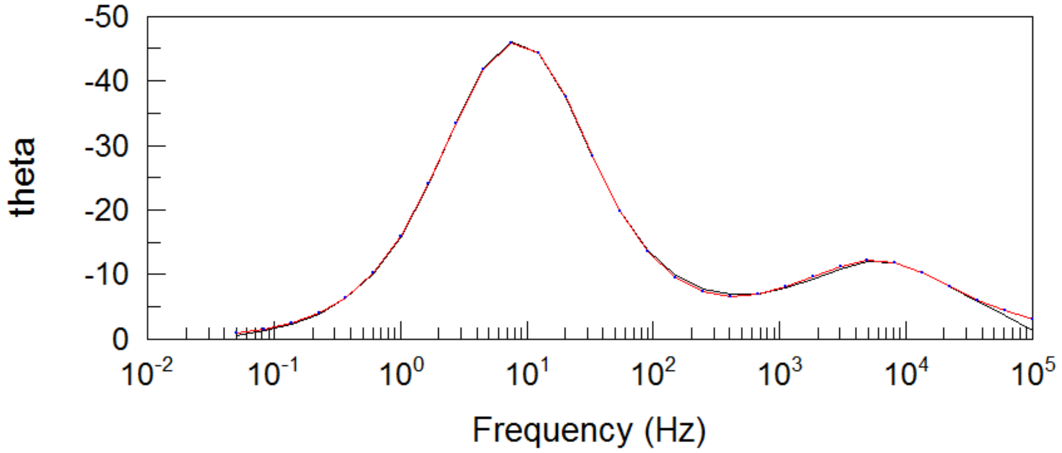

Nyquist (top) and Bode (bottom) plots of **E4**:

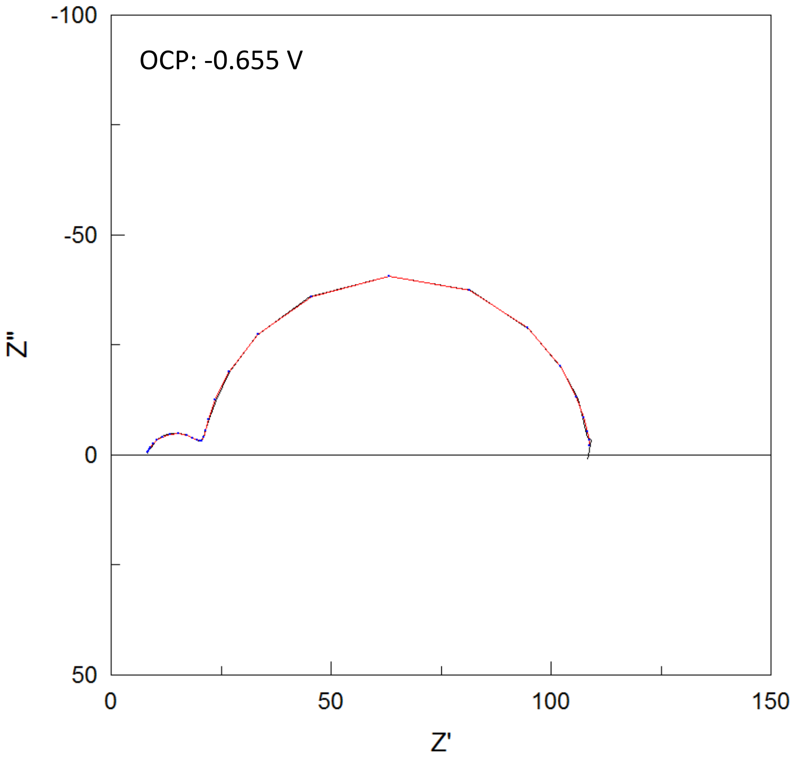

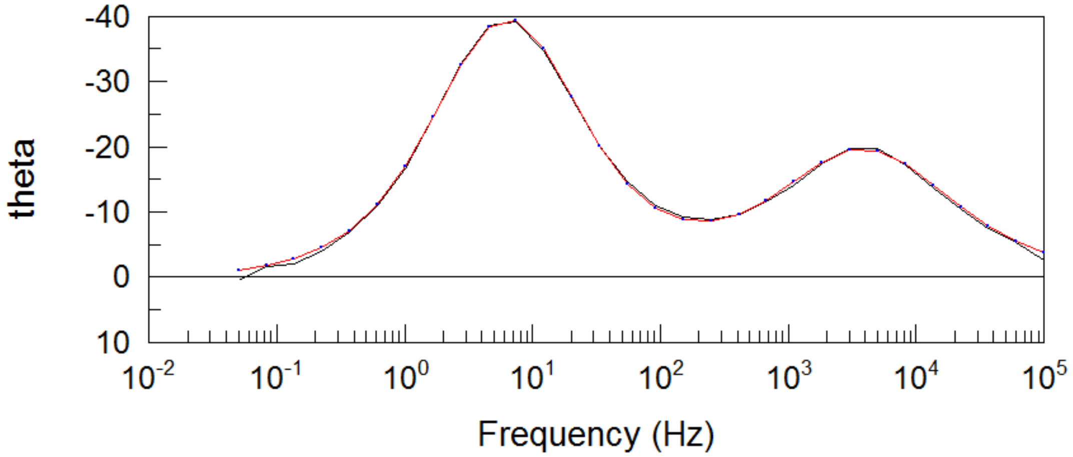

Nyquist (top) and Bode (bottom) plots of E5:

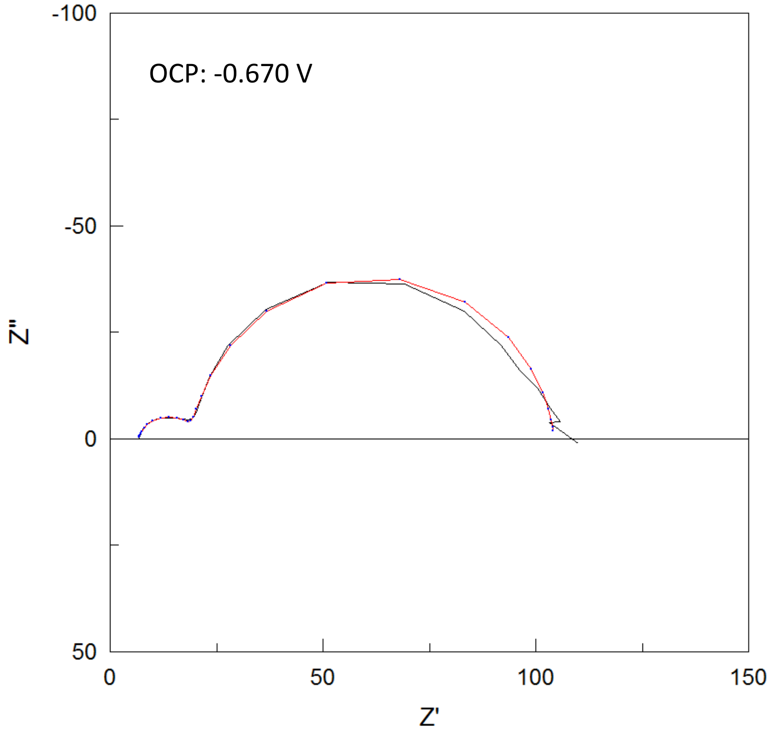

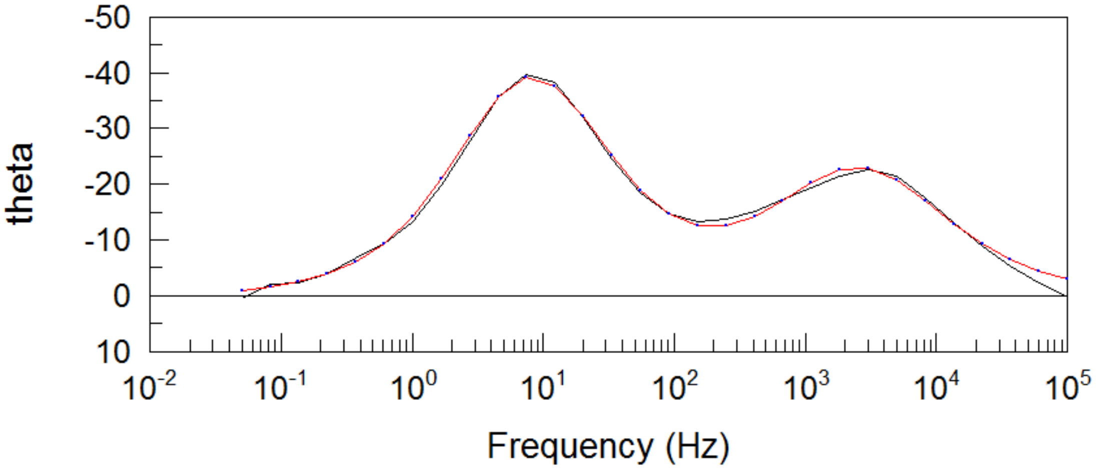

Nyquist (top) and Bode (bottom) plots of **E6**:

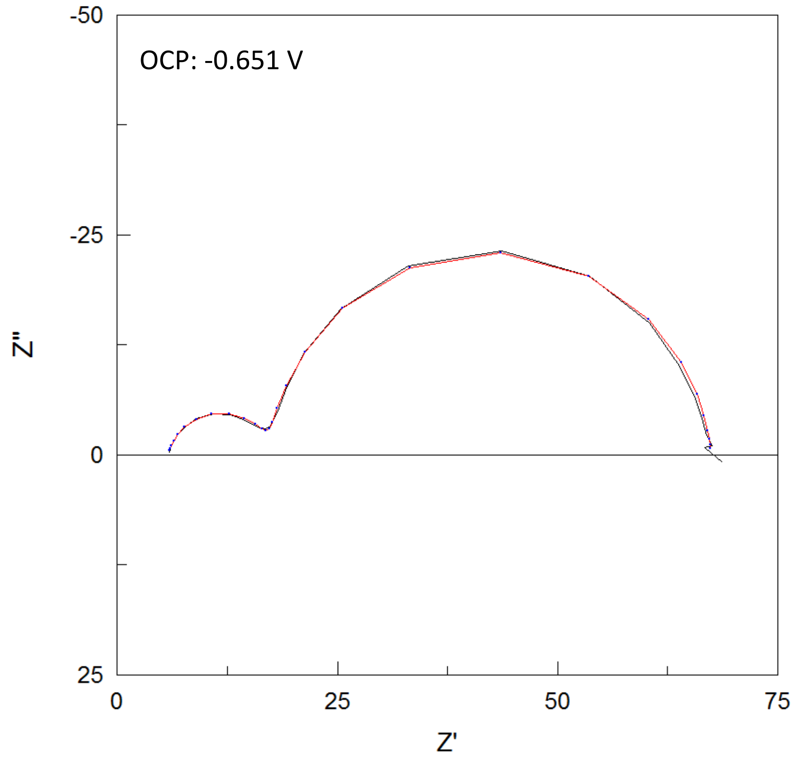

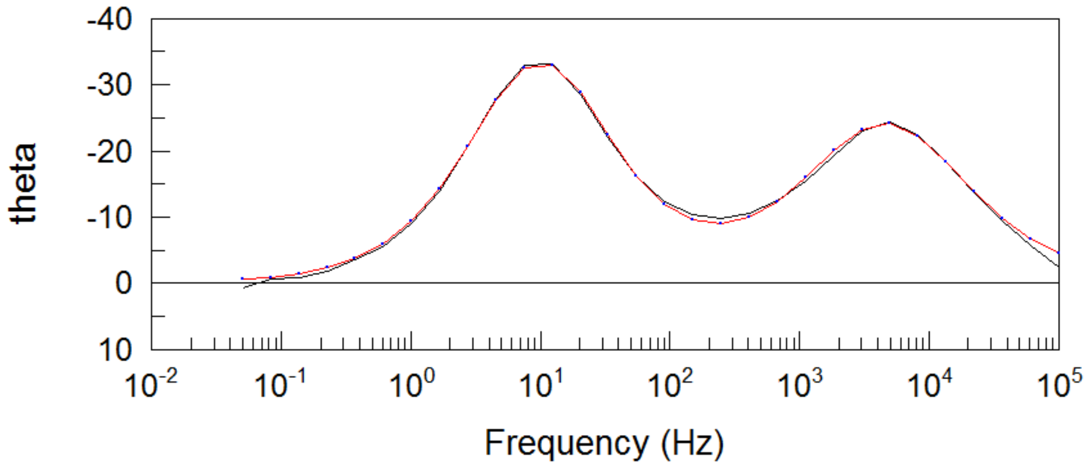

Nyquist (top) and Bode (bottom) plots of E7:

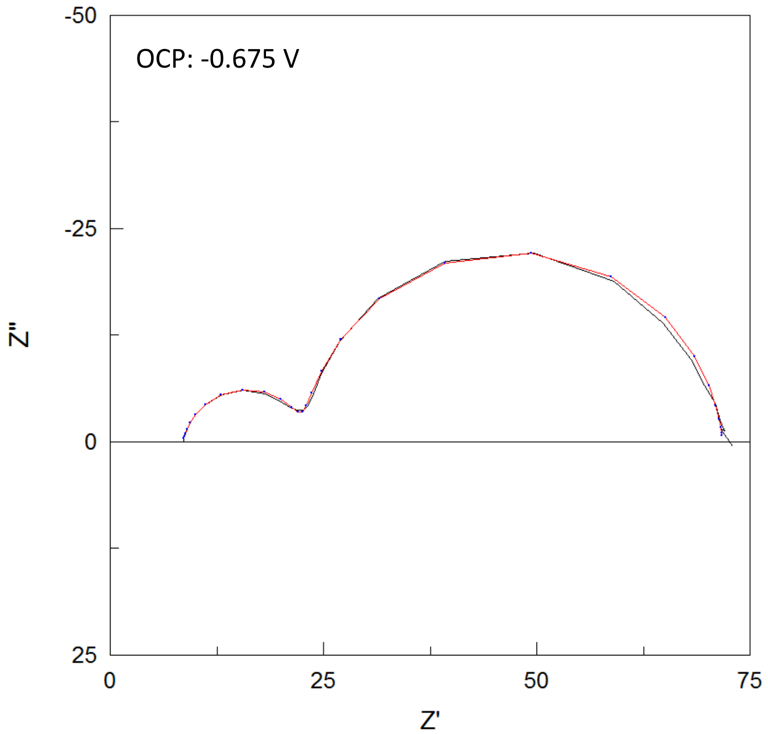

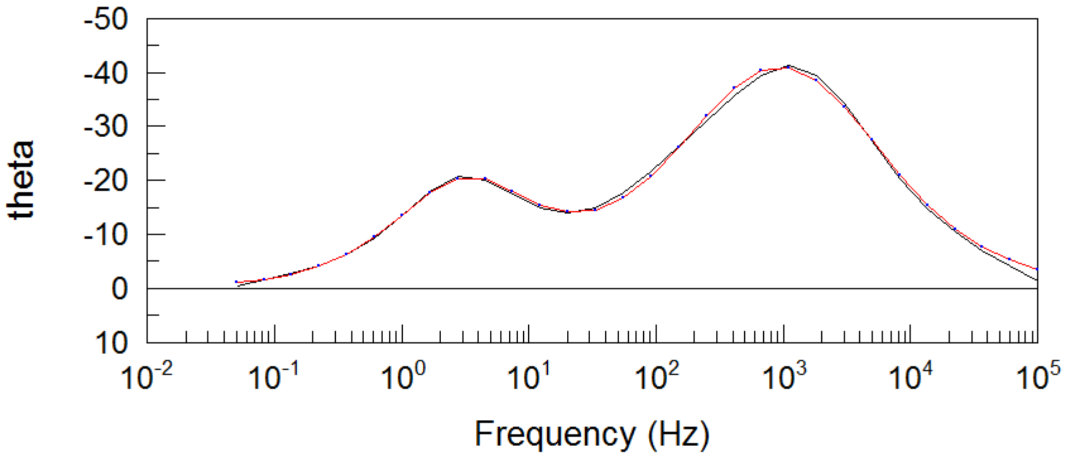

Nyquist (top) and Bode (bottom) plots of **E8**:

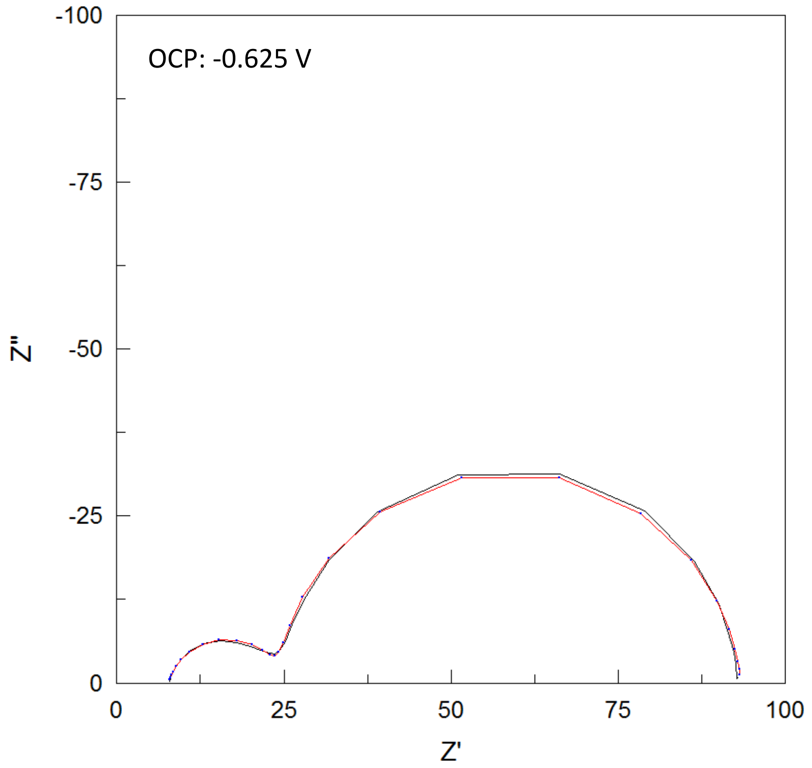

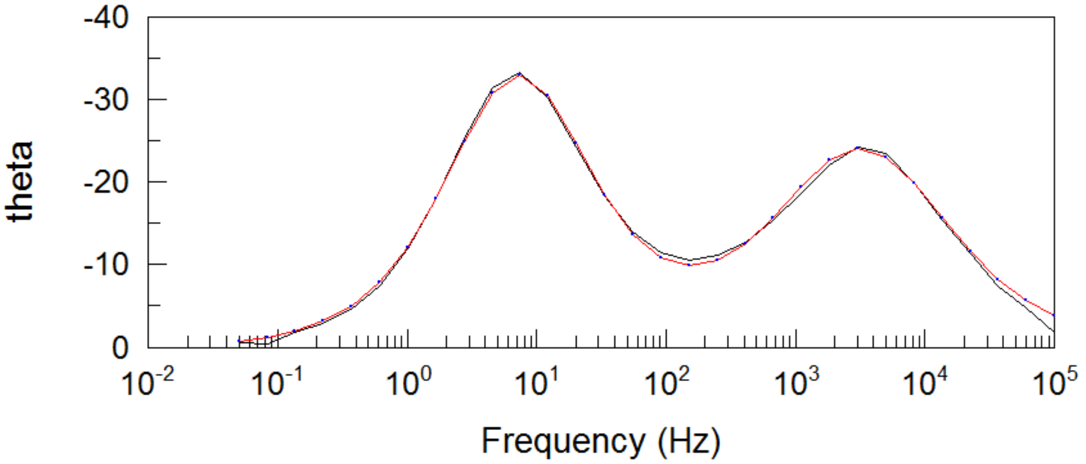

Nyquist (top) and Bode (bottom) plots of **E9**:

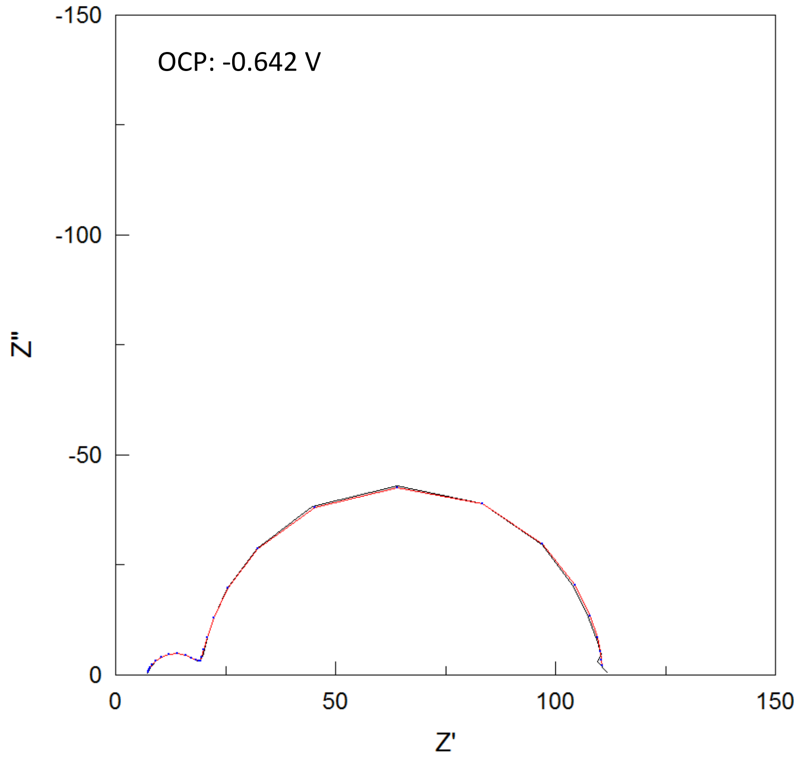

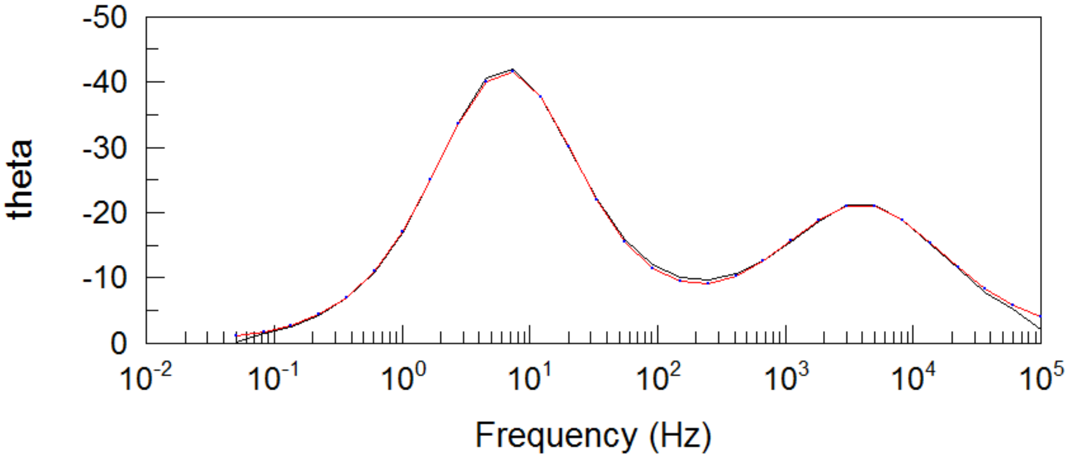

Nyquist (top) and Bode (bottom) plots of **E10**:

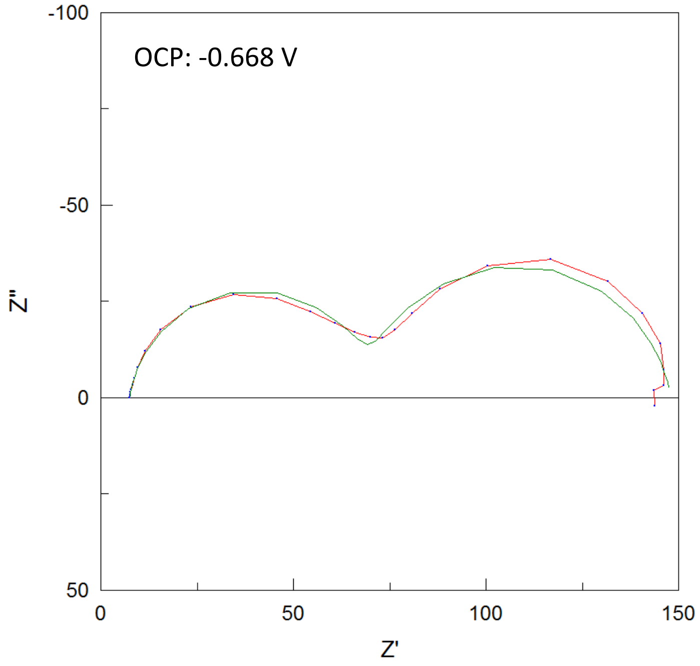

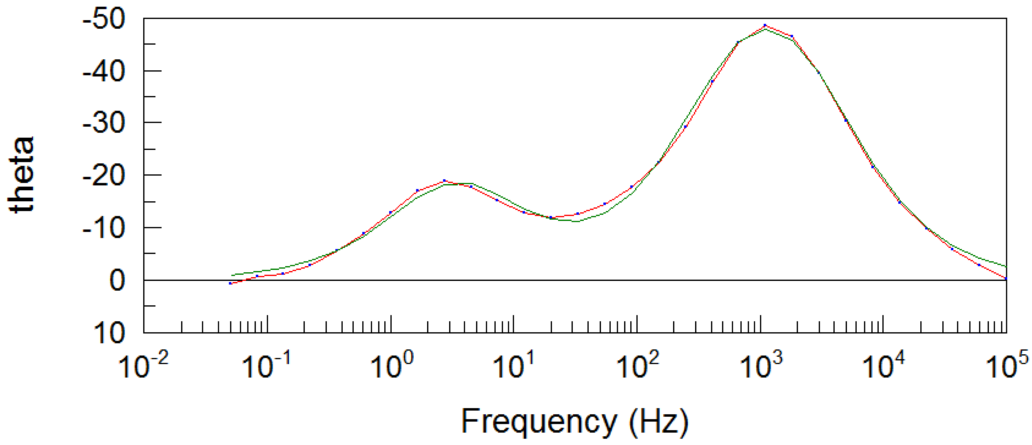

Nyquist (top) and Bode (bottom) plots of **E11**:

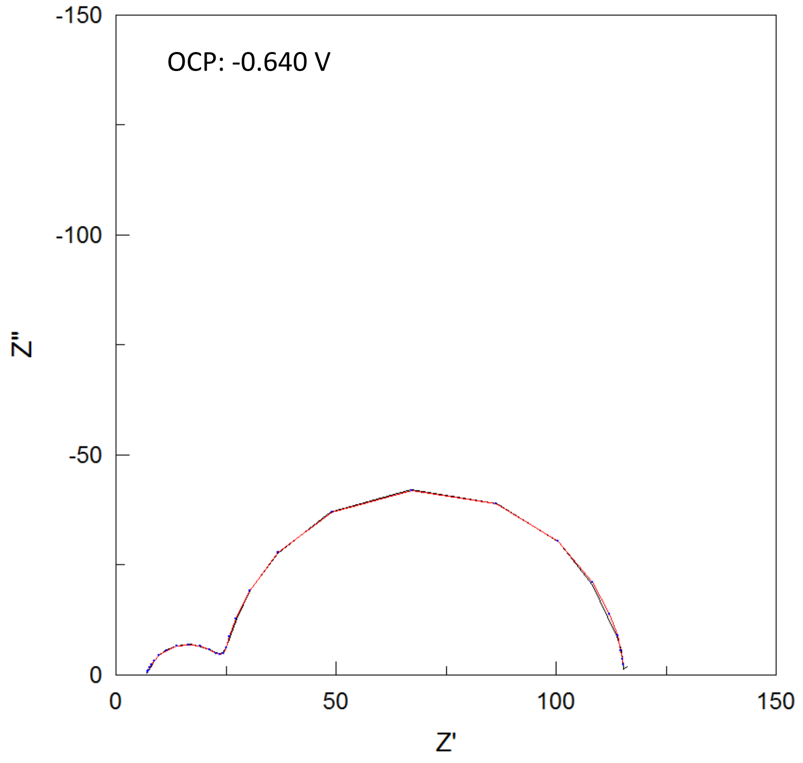

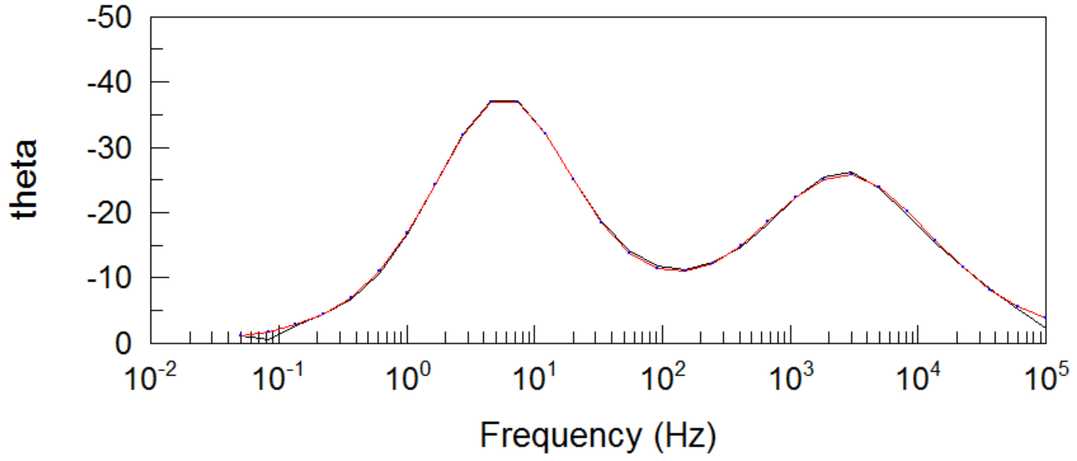

1. Huang, J.; Riisager, A.; Wasserscheid, P.; Fehrmann, R., Reversible physical absorption of SO2 by ionic liquids. *Chem. Commun.* **2006**, (38), 4027-4029, doi: 10.1039/b609714f.
